# Robust digital-twin airspace discretization and trajectory optimization for autonomous unmanned aerial vehicles

**DOI:** 10.1038/s41598-024-62421-4

**Published:** 2024-05-31

**Authors:** Mo ElSayed, Moataz Mohamed

**Affiliations:** Department of Civil Engineering, Faculty of Engineering, McMaster University, 1280 Main Street West, JHE Building, Room 301, Hamilton, ON L8S 4L7 Canada

**Keywords:** Autonomous UAV logistics, Resilient transportation infrastructure, Energy-efficient trajectory optimization, Aerial mobility operation model, Drones, Digital twin models, Civil engineering, Aerospace engineering, Computational science

## Abstract

The infiltration of heterogenous fleets of autonomous Unmanned Aerial Vehicles (UAVs) in smart cities is leading to the consumerization of city air space which includes infrastructure creation of roads, traffic design, capacity estimation, and trajectory optimization. This study proposes a novel autonomous Advanced Aerial Mobility (AAM) logistical system for high density city centers. First, we propose a real-time 3D geospatial mining framework for LiDAR data to create a dynamically updated digital twin model. This enables the identification of viable airspace volumes in densely populated 3D environments based on the airspace policy/regulations. Second, we propose a robust city airspace dynamic 4D discretization method (*Skyroutes*) for autonomous UAVs to incorporate the underlying real-time constraints coupled with externalities, legal, and optimal UAV operation based on kinematics. An hourly trip generation model was applied to create 1138 trips in two scenarios comparing the cartesian discretization to our proposed algorithm. The results show that the AAM enables a precise airspace capacity/cost estimation, due to its detailed 3D generation capabilities. The AAM increased the airspace capacity by up to 10%, the generated UAV trajectories are 50% more energy efficient, and significantly safer.

## Introduction

According to the United Nations, the world population is expected to reach 10.1 billion by 2100, cities are growing exponentially across the globe^1^. Given the limited space and resources, the concept of a smart city emerged, which is designed for the optimum usage of space and supplies along with an efficient distribution of resources. Smart cities are, by default, designed to achieve resilient communities that maximize the integration between humans and robotics^2^. Accordingly, the use of autonomous systems is considered a dire need to enable cities’ resilience and to cope with the economic, social, and environmental disruptions arising from expansions and increased population density. This has been highlighted recently with novel corona virus (COVID-19) pandemic, which necessitated quarantining and city lockdowns worldwide.

Autonomous systems’ integration in cities is featured in several applications such as robotic manufacturing, robotic construction, and autonomous transportation systems. Unmanned Aerial Vehicles or System (UAVs or UAS) or ‘drones’ are utilized in a variety of civil and military tasks such as cargo transport, emergency management, and search and rescue missions^3,4^.

This technological transformation is often equated to the paradigm shift created in automobiles by Henry Ford in the early twentieth century. In other words, the creation of roads, traffic design, and planning can be applicable to the consumerization of city air space. While UAV applications are relatively easier in rural areas, however, several challenges arise with the anticipated proliferation of heterogenous UAV fleets in low-altitude airspace of dense urban areas given characteristics of cities and the definition of relevant decision variables^5^. These challenges can be bundled according to the elements of the autonomous UAV system, the UAV itself, and the city airspace as the hosting infrastructure.

As it relates to the UAV, and to maintain the overall weight of the UAV, a trade-off is inevitable between the on-board power source, processing unit, and sensors essential for autonomy, and the transported payload limits and/or the flight range. This trade-off decreases the practicality of the whole independent UAV system^6^. Significant advancements in UAV technologies promise an increased energy efficiency, lighter airframes, and improved power-to-weight ratio for DC motors. However, these improvements are not expected to reflect substantially on the existing performance in the near future^7,8^. Therefore, research has depended on developing routing algorithms or trajectory planning and optimization heuristics to tackle current UAV limitations^9^. Most relevant to low altitude airspace management (LAAM) applications is a UAV Routing and Trajectory Optimization Problem (RTOP), where a fleet of UAVs visits a set of waypoints (missions) assuming UAV kinematics (position, velocity, and acceleration), and dynamic (forces and moments) constraints. This represents half of the solution, since it depends on the presence of a viable discretized airspace that respects all other constraints.

Considering city airspace challenges, different variables exist. First, massive fleets of UAVs operating in highly dense cities raises serious safety issues as huge damage can be sustained to pedestrians and public or private property. This damage can be caused by the crashing of a UAV due to a technical malfunction or mid-air collisions due to airspace interference and congestion^10–12^. Second, UAV onboard communication and GPS navigation modules are vulnerable to security breaches due to the unencrypted nature, which makes it easily spoofed^13,14^. Third, given their data collection abilities, sensors, and high-precision onboard cameras, UAVs can be perceived as remotely controlled surveillance equipment^15^ as they can be hacked to collect personal data or track individuals using wireless localization techniques. Fourth, the proximity to public operation causes pedestrians to feel uncomfortable or dwellers to feel that their privacy is being invaded^3^.

Given these challenges and the traditional concepts of city security, liability, and aviation airspace regulations, the need to regulate UAV operation pushed international, federal, and local governments to navigate unchartered territories, with boundaries of civil regulatory authority over UAVs ill-defined^12,16^. Currently, several countries have imposed UAV operational restrictions based on proximity to population and man-made structures. While these regulations alone can control leisure UAV use, however, heterogenous fleet operation with projections of massive volumes of UAVs is too large for the current Air Traffic Control structure to handle^17,18^.

Reacting to that, two types of research exist, one group focusing on enabling safe urban airspace operation through geofencing and airspace discretization or air traffic control. The other group works on developing routing algorithms or path/trajectory planning heuristics for conventional cartesian airspace. While some of the previous research consider some of the aforementioned challenges/parameters, others fall short in providing comprehensive applicable frameworks/solutions^3,4^. The adoption of UAVs in autonomous transportation within smart cities hinges on the development of a full city aerial-infrastructure framework for operation (airspace discretization and geofencing), navigation (trajectory planning), and traffic control of swarms of UAVs running on robotic operation systems (ROS)^19^. This framework must consider all challenges raised by previous studies across all parameters before proceeding into a real-life execution phase.

In this respect, the present study develops a novel autonomous Advanced Aerial Mobility (AAM) system for high density city centers. The AAM system integrates a digital-twin city-airspace discretization, planning, and trajectory optimization algorithm for heterogeneous UAV fleets.

To the authors’ knowledge, this is the first study to integrate live, updated, precise digital models with airspace planning for exterior complex urban environments. An extensive review of the most recently developed methodologies integrating GPS data and LADAR for UAV pose estimation and trajectory is provided by Vaidis^20^, and the latest LiDAR 3D processing techniques by Wang et al.^21^. This also is the first study addressing the intertwined city airspace regulatory challenges and the multiple parameters for efficient UAV operation within digital-twin models. The primary aim of this study is to develop a novel comprehensive algorithm that allows autonomous AAM operation within civil airspace. The model depends on dynamically updated real-time LiDAR data to simulate the actual civil airspace and converge energy-efficient pre-planned obstacle-avoiding trajectories instead of active path planning for each UAV. The proposed system solves airspace planning and UAV control/navigation challenges, accommodating variable UAV sizes, types, and speeds. Furthermore, ensuring abidance to respective airspace regulations and maximizing capacity.

To achieve this aim, the present study,

1. Develops a real-time 3D geospatial mining framework for geo-referenced allocation of trips and UAV task assignment based on LiDAR data to create a digital twin model.
2. Proposes a novel city airspace dynamic 4D discretization method (Skyroutes) for UAVs based on legal regulations incorporating real-time constraints coupled with external factors. The discretization converges a network of keep-in lanes allocated outside the keep-out geofence (dual geofencing).
3. Utilizes a flexible energy use model for multi-rotor UAVs based on the kinematics and dynamic operational capabilities and calibrated to measurements from representative experimental UAV flights^22^.
4. Develops a dynamic trajectory optimization method tailored for the proposed discretization method coupled with a novel 3D lane-change and compare the solutions’ efficiency to the existing algorithms in the literature.
5. The developed models are applied to a real-world case study to computationally simulate UAV transportation operations delivery applications.

In this study, after presenting the airspace discretization model, we formally define the UAV routing and trajectory optimization based on quadcopter kinematics and dynamics. We utilize Newton–Euler derived differential equations to simulate the operation of UAV brushless DC rotors. Thereafter, we utilize a complementing simplified real-time Dynamic Programming (DP) arc-routing method to determine minimum Snap and energy trajectory for a fleet of UAVs visiting a set of arcs between origin locations and destinations. In this respect, the presented study provides an original contribution to the AAM challenge.

After this introduction, a literature review focusing primarily on different approaches to UAV-city-integration through civil air-space discretization and UAV trajectory planning research is presented in “Literature review”. “Methods” introduces the study methodology, while “City digital twin model” and “Airspace discretization model description” include the Digital-twin model and the proposed urban airspace discretization derivation model respectively. The modified energy-optimal trajectory planning and UAV task assignment framework are detailed in “Trip generation, Cartesian routing, and UAV energy consumption”. “Case study, results, and discussion” reports on the case study, the results, and the discussion, while conclusions are presented in “Conclusions and future studies”.

## Literature review

Currently, UAVs’ operation is limited below flying altitude of commercial aircraft to avoid collision potential. Globally, this can be generally defined as zero to 150 m over ground level^23^. Although autonomous UAV mission control can be performed onboard with the reliance on sensors, GPS, and computation. However, in proximity to buildings and in case of severe weather conditions, UAVs are prone to loss of GPS signal or sensor failure jeopardizing the efficiency and stability of the entire network^24,25^. Hence, off-board preloaded mission planning maximizes safety, utility, reliability, and mitigates the need for onboard multiple sensors saving weight for payload and decreasing costs. Autonomous operation within densely built-up areas could interfere with UAVs^26^.

In response to that, earlier research on LAAM recommended the development of a unified urban airspace system or ‘urban air mobility,’ to manage the safe operation of UAVs within low-altitude civilian airspace. For instance, in 2006, the International Civil Aviation Organization (ICAO) declared the need for international harmonized terms and principles to guide the civil use of UAVs^27^. Later in 2020, the term Advanced Aerial Mobility (AAM) was coined by NASA denoting the ecosystem incubating the emergence of these disruptive technologies in both urban and rural contexts^28^. The published report outlines a vision for the city airspace and air traffic management environment.

The main concept is to establish a national framework through a unified infrastructure with levels of complexity for all manned and unmanned aerial vehicles of any size or type to control traffic, separation, and flight trajectories. The airspace traffic network is to be based on data sharing and utilized as a nationally controlled utility provided for various mobility operators similar to current ground road networks^28^. To that end, the economic, social, and regulatory success of the system is dependent on addressing some fundamental challenges which can be summarized as follows:

- Safety and security: any AAM must ensure the safety of public and private property and users such as collision avoidance, limiting extreme proximity and mitigation of street level drivers’ visual distraction. In addition, including cybersecurity against communications signal hacking beside other system vulnerabilities in extreme weather conditions and disruptive events^29,30^.
- Environmental impacts: this entails minimizing or eliminating GHG emissions, noise, and impact on wildlife^22^.
- Flexibility and resilience: the system’s ability to recover quickly from unexpected events and limit the cascading impact. Furthermore, the ability of the system to evolve with the emergence of newer UAV technologies, software, and operational concepts^31^.
- Regulation: develop standardized national policies to govern operation and allow insurance and tax or toll collection.
- Social acceptance: being a disruptive technology with a social stigma, experimental real-world operations and scenario-based analyses can convince the users that the urban obtrusiveness risk is acceptable, and efficient to overcome cost barriers.

To tackle these challenges, over the last decade ample research has aimed to provide solutions or guidelines, those can be bundled in two groups according to the targeted study area, airspace planning research and UAV navigation and control research.

### Urban airspace planning

The concept of airspace planning as explained from the AAM perspective and challenges is new to the research community^28^. However, a substantial part of this area is commonly researched under the UAV Traffic Management (UTM) keyword, where ample literature exists^32,33^. With that said, only literature on obstacle-rich lower urban airspace is considered in this review rather than obstacle-free higher-altitude airspace.

The most researched concept of separating flyable airspace from obstacles in UTM is known as Geofencing^34^. Geofence is a virtual perimeter applied statically or dynamically in a real-world application in positive (keep-in) or negative (keep-out). While the keep-in geofence is a 3D volume to maintain, the keep-out is applying volumetric restriction to certain extents. With current UAV regulation generally including a minimum distance or protection boundary around static objects (e.g., people, buildings, and structures) and altitude limit^23^, keep-out is the most deployed and researched methodology^35^.

Urban airspace planning depends on two factors, namely the quality of the 3D environment model and the geofencing technique utilized. The accuracy of estimating a real-time state of UAVs is highly dependent on digitally replicating the real-world environment. This relies primarily on the collected surrounding sensor data. Literature has mainly depended on 3D GIS, Digital Surface Model (DSM), or Google’s 3D city data for their system simulations^36–38^. While 3D maps provide viable results, they fall short to include details and dynamic changes to the real-world environment. The missing details and changes include transmission towers, utility poles, power lines, construction equipment including cranes, and street level vegetation.

**Figure 1:**
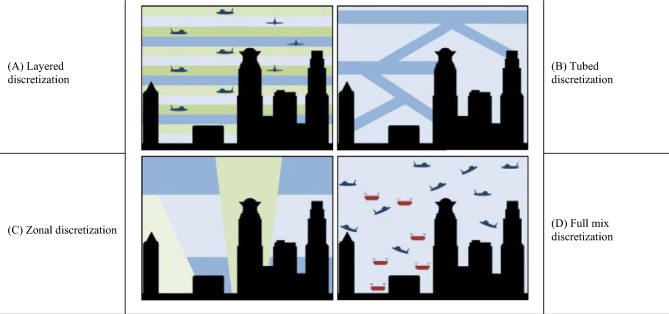
Different airspace discretization morphologies^36^. Source: Patrinopoulou et al., 2022.

The integration of airspace planning and geofencing has been studied under various discretization morphologies^37–41^. Most comprehensively, Hoekstra et al.^36^ illustrate all the discretization morphologies (Fig. 1). In all morphologies, on‐board avionics implement the preloaded regulation and specific flight plan autonomously, and the flight trajectory is governed through the geofence.

Another stream of studies targeting specific challenges exist in the literature. Although they do not provide holistic solutions, however, their conclusions and recommendations are exceptionally valuable for building on towards the objective of this study. D’Souza et al.^42^ tested the flight deviation from planned path due to wind disturbances. Their study concluded that PID controller stabilization can decrease the minimum lateral distance from buildings up to 5 m. Similarly, Johnson et al.^43^ tested applying several minimum lateral distance alternatives on the ability of UAVs to detect and avoid buildings. Their results showed poor detection capabilities with narrow urban corridors. Recently, Cho and Yoon^44^ compared three scenarios for the case study of Seoul city, namely keep-out, keep-in, and dual geofencing. They concluded that keep-in exhibited more robust behavior than the keep-out. The study recommended integrating both geofencing methods while applying dynamic parameters given the geospatial complexity and flight purposes. More recently, Torija et al.^45^ compared a series of audio-visual scenarios for UAV operation in cities to investigate the impacts of UAV noise. They concluded that the UAV operations along busy roads might aid in the mitigation of the overall community noise impact.

Overall, the concept of AAM has been mirrored globally in numerous studies with the aim of establishing a comprehensive UAV airspace discretization framework. However, most research has focused on the integration of one or two challenges rather than addressing all challenges.

Table 1 presents a summary of the most relevant literature outlining the solutions and recommendations for each challenge. Although several other studies address the same topic, the following limitations were applied in the filtration process. (1) The oversimplification of the problem, which makes the solutions less robust for city-scale application. (2) Proprietary restrictions that prohibits the open collaboration on developing, integrating, and testing the suggested solutions^18^. (3) Solutions not targeted for autonomy and beyond visual line-of-sight (BVLOS), since solutions for piloted systems are significantly different and not viable^46^.

**Table 1:** Urban airspace planning literature.

Project	Safety and security	Environmental impacts	Flexibility and resilience	Regulation	Social acceptance
Metropolis project^36^	Full mix, layered, zonal, and tubed	Optimal route geometry	Flow management, separation, and conflict avoidance	Considers only average regulation strictness	Presenting alternative scenarios
	Limited static airspace planning in two models. Exclusive limitation of operation within obstacle-free airspace decreases mission range for smaller UAVs and increases mission time for emergency application				
Singapore’s TM-UAS^37,38^	Full mix (AirMatrix), over buildings, and over roads	N/A	Flight and risk management	Varying degrees of regulation	Presenting alternative scenarios
	Limited static waypoint concept. Limited route optimization and flexibility with UAV types and sizes given only constant speed or random speed optimization limitation in traffic control				
Australia’s Smart Skies project^39^	Automated separation management system	N/A	Sense-and-Act Systems	Local current regulation	N/A
	Limited model details. Limited route planning and optimization capabilities through the developed Mobile Aircraft Tracking System (MATS)				
NASA UTM^40,41^	Collision avoidance	N/A	Contingency management and re-routing	FAA regulation	Public safety illustration
	Limited multi-modal airspace discretization and modification				

### UAV flight navigation and control

Given a fleet of UAVs and a volume of designed urban airspace, the actual navigation and mission control can be described as a series of complex mathematical problems. First, the NP-hard UAV Task Assignment (TA) problem refers to optimally assigning missions to a set of UAVs based on mission constraints^47^. While TA shares similar characteristics with Vehicle Routing Problem (VRP), there are a few key differences^48,49^. Unlike VRP, TA allows multiple stops, heterogeneous fleet operation, and mission subtours. The output for both VRP and TA is a pairing between a set of O-Ds, and assigned vehicles along with a set of waypoints.

Second, to connect these waypoints and form a UAV flyable path, the problem is known as Path Planning (PP). PP is defined as the process of constructing a geometric path from a starting point to an end point given a 2D or 3D domain. While PP can include the impact of wind or other constraints, the problem formulation is extensively simplified to be solved heuristically^50^. In a real-world application, it is imperative to couple the generated path with UAV constraints, kinematics, and dynamics^51^. In this respect, the integration of kinematics and dynamics with routing is known as optimal motion (trajectory) planning or Trajectory Optimization (TO). Closely related to the Optimal Control (OC) problem, TO leverages motion equations to model the spatiotemporal changes of the UAV system while minimizing a scalar performance index such as flight time or fuel consumption^52^. A detailed literature review of the research focused on these problems can be found in the literature^19^. They conclude that on one hand, the UAV routing and task assignment literatures have mostly neglected complex UAV constraints. On the other hand, TO research has fallen short in integrating other noise and safety challenges.

Research about integrated routing and trajectory optimization is scarce^19^, however, there are ample studies discussing different methods for UAV navigation and mission control. Literature can be classified based on methodology into three major groups. (1) Mixed-Integer Linear Programming and exact algorithms (such as branch and bound or Euclidean minimum spanning tree), where an optimal solution is guaranteed; (2) Metaheuristics such as Evolutionary algorithms, Particle Swarm Optimization, and Ant Colony, where a solution is not guaranteed; (3) Heuristics that includes merging different heuristics or special cases of algorithms such as hybrid Tabu Search-Simulated Annealing^53^. Several other methods are presented in the literature without belonging to a specific group. To focus on relevant literature, we only present in Table 2 studies that can perform combined routing and trajectory optimization independent of urban airspace planning. Therefore, obstacle avoidance (static or dynamic) and 3D environment operation are imperative capabilities^54^.

**Table 2:** Relevant UAV 3D routing and trajectory optimization literature.

Method/author(s)	Method	Application	Simulation/experimental verification
Trajectory optimization of multiple quad-rotor UAVs in collaborative assembling task^55^	Genetic algorithm	Uncapacitated Multi UAV trajectory optimization	Simulation
	No real time applicability for heterogeneous fleet/swarm operation. No wind consideration		
3D off-line path planning for aerial vehicle using distance transform technique^56^	Multi criteria decision analysis	Off-line path planning	Simulation
	No real time applicability for heterogeneous fleet/swarm operation. Limited UAV dynamics accountability and lacking wind consideration		
A heuristic mission planning algorithm for heterogeneous tasks with heterogeneous UAVs^57^	Heuristic algorithm	Mission planning for heterogeneous tasks	Simulation
	No real time applicability and wind consideration. Limited UAV dynamics accountability		
3D multi-constraint route planning for UAV low-altitude penetration based on multi-agent genetic algorithm^58^	Genetic algorithm	Mission multi-constraint route planning	Simulation
	No real time applicability for heterogeneous fleet/swarm operation. Limited UAV dynamics accountability and lacking wind consideration		
Distributed pseudolinear estimation and UAV path optimization for 3D target tracking^59^	Gradient-descent algorithm	UAV path optimization for 3D target tracking	Simulation
	No real time applicability for heterogeneous fleet/swarm operation and wind consideration		
Online path planning for UAV using an improved differential evolution algorithm^60^	Differential Evolution Algorithm	Online path planning for UAV	Simulation
	No real time applicability and wind consideration. Limited UAV dynamics accountability		
Trajectory planning for unmanned aerial vehicles in complicated urban environments: A control network approach^61^	control network and Dubins curve Algorithm	Two-stage control network approach	Simulation
	No applicability for huge-scale cities, the control network could contain billions of links and it may cause the path-finding problem computationally burdensome/over-simplification of the city model		
3D path planning and real-time collision resolution of multirotor drone operations in complex urban low-altitude airspace^62^	3D voxel jump and Markov decision process	Autonomous drone collision-free path planning	Simulation
	Originated from the classical 2D grid map JPS method considers only diagonal or straight directions/over-simplification of the city model		

To that end, while limitations of case-by-case studies in the literature can be addressed, all routing and trajectory optimization methods presented utilize a cartesian discretization rather than incorporating a discrete airspace planning and discretization component leading to a full-mix airspace concept. In this concept, airspace is unstructured and UAV traffic is fully dependant on onboard sensing, self‐regulation, and obstacle avoidance under stochastic conditions. Although the full-mix airspace concept allows for maximum speed and freedom, given heterogenous fleet operation, it severely limits the energy efficiency, airspace capacity, as well as jeopardizing system‐wide safety. Hence, a full-mix concept fails to address the aforementioned AAM challenges^22^.

The innovation in this study diverges from existing work primarily through its dynamic 4D discretization method known as “Skyroutes” , which contrasts with traditional Cartesian discretization methods. In lieu of Table 2, Skyroutes is designed to be open-source and incorporate real-time constraints along with legal and optimal UAV operation considerations based on kinematics. This allows accounting for wind consideration. Furthermore, the simplified computational burden allows for real-time applicability to a heterogeneous fleet/swarm operation. Prior relevant studies have relied on 3D GIS Digital Surface Models^63–65^ or similar data sources for system simulations^60–62^, which, while effective, do not account for dynamic changes to the real-world environment and often lack the details necessary for accurate urban airspace planning.

Integrating real-time, high-resolution digital twins in the proposed AAM system represents a significant advancement in urban airspace management, providing a more accurate and dynamic method for airspace capacity estimation and UAV trajectory optimization. This system not only improves upon the limitations of existing approaches with its detailed and adaptive modeling but also demonstrates the potential for increased airspace capacity and safety in urban environments. The novel Skyroutes discretization method, in particular, represents a key differentiation from traditional methods, offering a more efficient and flexible solution for airspace management in densely populated urban settings. The proposed system is not a stand-alone platform. However, it could be applied within a platform similar to the European U-Space^66^.

## Methods

**Figure 2:**
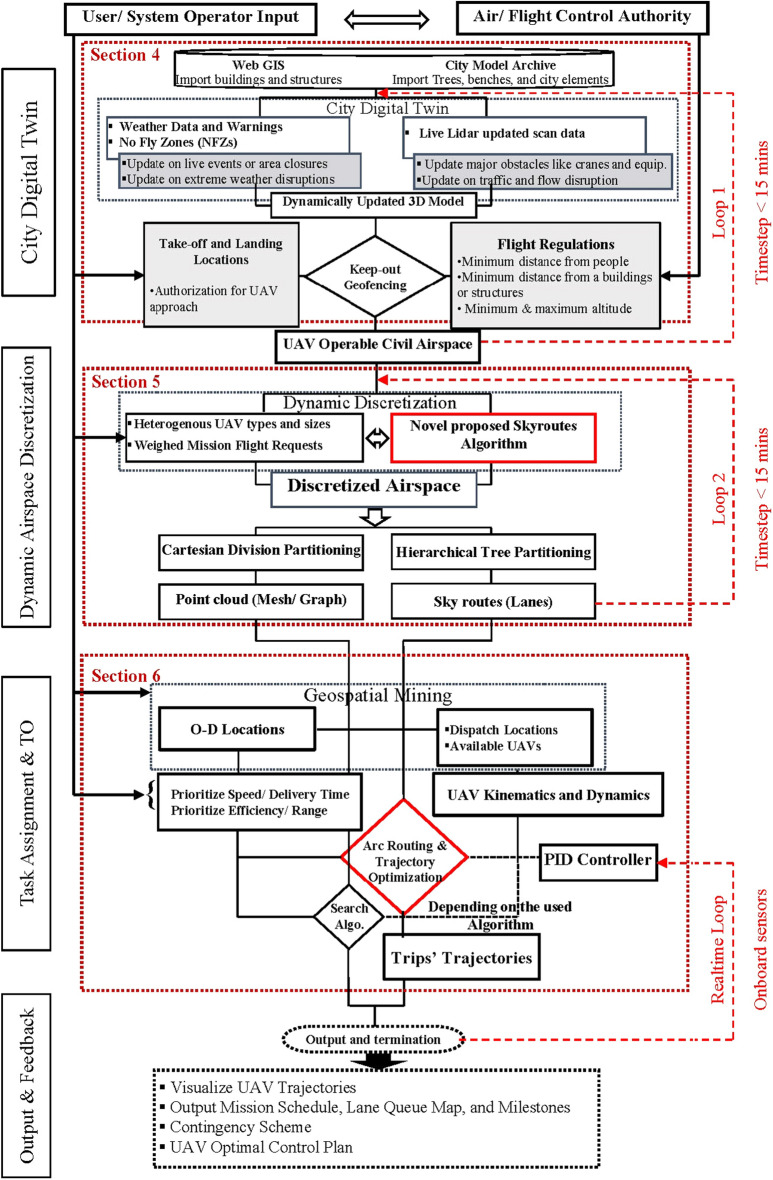
The developed methodology.

The study proposes a novel three-step sequential methodological approach (Fig. 2). Each step is detailed in the following sub-sections. In the first process, a digital-twin for the simulated case study is built and actively updated for real-time changes and disruptions.

Subsequently, the system obtains two streams of input for a set of variables through an online connection. The first stream of input relates to mission planning while the second stream relates to the area-specific flight regulations adopted. Using a keep-out geofence, all geometry is interpreted into physical obstacles. The second process starts by sorting the heterogenous input UAV and applying our proposed novel *Skyroutes* algorithm to produce a discretized airspace. This triggers the third process for task assignment, routing, and trajectory optimization. The fourth process is the final system output including the maximum airspace capacity. At the end of the procedure, the framework visualizes the UAV trajectories and provides the trajectories. An active loop is initiated between the UAV proportional–integral–derivative (PID) controller to correct the trajectory navigation as the operation progresses.

## City digital twin model

Detailed spatial information infrastructure crucial for the AAM system, however, it should be lightweight enough for the Ground Control System (GCS) and Central Control System (CCS) to handle in real-time. Given the number of details in urban environments and spatial approximation of object-based 3D information pose significant challenges on computational power and time. In this study we only require a level of tolerance < 1 m excluding take-off and landing, which is a function of onboard sensors and under slow flight speeds.

**Figure 3:**
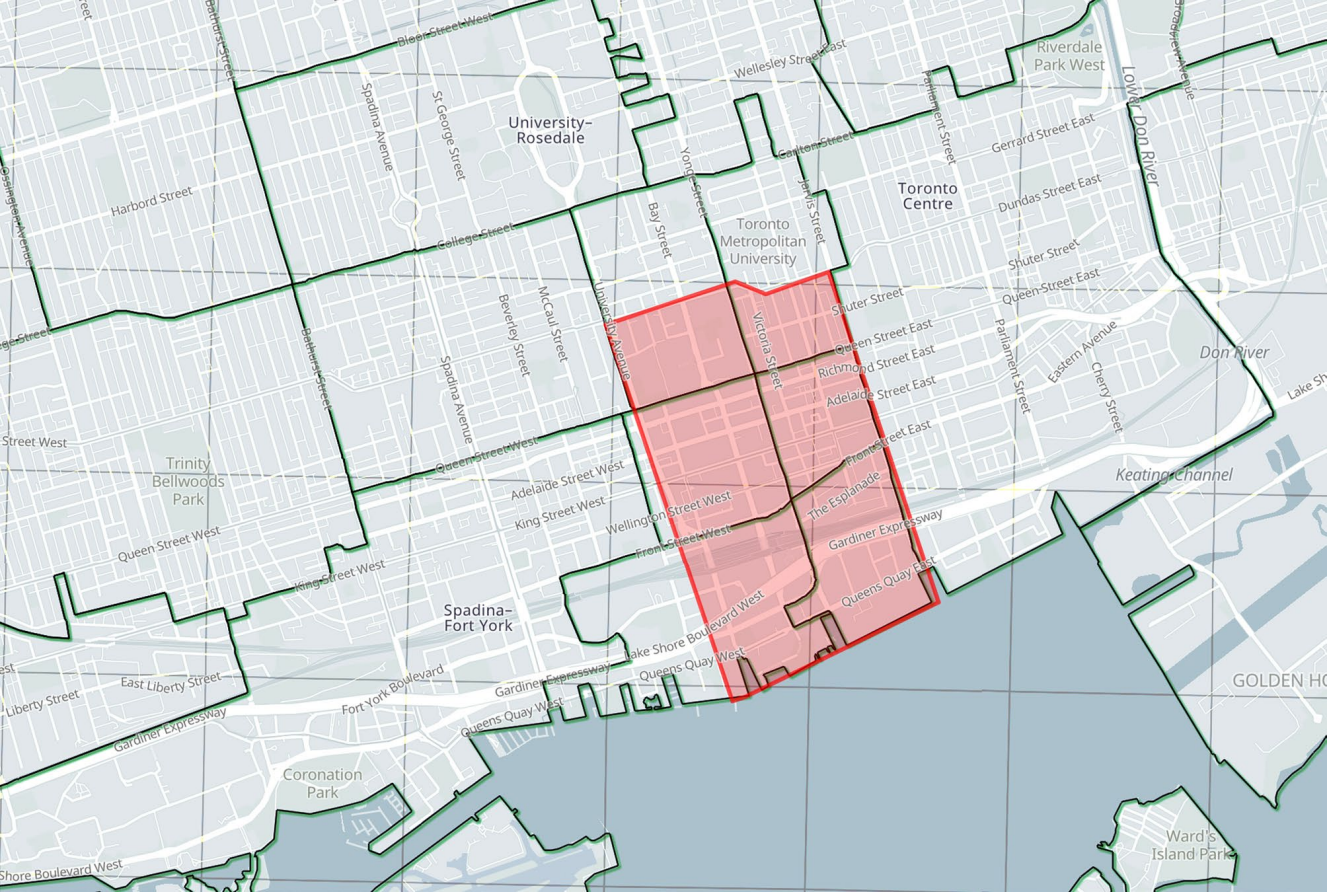
Overlay of 3D data sourcing, Blue (municipal), Green (postal), Black (lidar).

The city is divided into small bands using a 3D clustering method proposed by Youn et al.^67^ to maintain the memory consumption and computational power within viable limits. Their UAV 3D clustering proposes a 20-level grid division. Having equal division possess two challenges, first, the obstacle details are not equal comparing urban to rural contexts, which leads to computing memory unbalance. Second, the synchronization between this independent classification versus existing addressing and GPS positioning system adds a layer of computationally demanding processing. We modify their clustering system via 3-digit postal code classification to ease the geo-referencing with existing census population density for trip generation (Fig. 3). Further explanation of this partitioning scheme is beyond the scope of this study.

For each selected airspace planning zone, first, DSM is imported and overlaid to the OpenStreetMap (OSM)^68^ which acts as the base map that includes the vector data for precision 3D GIS alignment. The GIS map includes most data layers such as streets, zones, functions, and property outlines. Second, to Incorporate vertical building façade details (windows and balconies), municipal open-data environment is imported, scaled, and georeferenced in the simulation model. Finally, most recent real-life LiDAR data is merged in the model for interpolation and updates to make sure the digital-twin model can truly reflect the reality once UAVs are deployed in a large scale.

Since LiDAR data are characterized by noisy patterns due to errors and the complexity of surfaces, these datasets require further processing to be usable for discretization. A variety of 3D extraction algorithms is discussed in the literature^21^. For the purpose of airspace planning and navigation for UAVs, there is no need for distinction between urban elements such as buildings and trees., (i.e., the objective is to avoid all obstacles). In this study, we modify a Freeform objects reconstruction algorithm via Poisson method proposed by Kazhdan et al.^69^ instead of a topographical or building extraction algorithm. The Poisson method is widely popular due to its scalability and efficiency where it can reconstruct freeform objects fast and with reasonable accuracy^21^. The solution Eqs. (1–4) are outlined in the appendix as they are auxiliary to the research question.

At this point, we have attained realistic iso-surfaces to construct a mesh of the existing real-world city environment. However, to comply with regulations, the horizontal distance or protection boundary around objects must be added to the obstacles model. Therefore, the model is shifted by a distance *δ*_*o*_ to offset obstacle meshes outward according to the applicable flight regulative distance. This is a direct application of the mathematical problem known as constant-distance offset (CDO) or specifically Minkowski sums for 3D geometries. Since the dataset contains complex non-convex polygons, we overcome this by utilizing a modified 3D scaling algorithm to shift each boundary representation (Brep)/ mesh face with the exact policy enforced distance. The Minkowski sum of two geometry sets $A$ and $B$ is defined as $A\oplus B = \{a + b | a \in A, b \in B\}$. If we take $A$ to be the arbitrary input mesh and $B$ a sphere of the given radius equal to policy-driven value *δ*_*o*_ centered at the origin, then an offset surface is defined as the boundary of their Minkowski sum. Detailed mathematical formulation for solid offsetting can be found in the work of Rossignac and Requicha^70^.

Given the city’s complex highly detailed polygonal mesh, each obstacle boundary *O* with Brep/mesh faces interpolates polygons ${\int }_{o}^{e}{\overline{p} }_{o}$ = ($x, y, z$) = [(${x}_{1},{ y}_{1}, {z}_{1}$), (${x}_{1}, {y}_{1}, {z}_{1}$), …, (${x}_{w},{ y}_{w}, {z}_{w}$)]. The new shifted faces are prescribed by set of vertices, *υ''* = ($x{\prime}{\prime}, y{\prime}{\prime}, z{\prime}{\prime}$). However, the new offset boundaries’ sum can include parts of spheres, cylinders, and prisms corresponding to vertices, edges, and faces of the mesh, respectively. To have closed Brep suitable for Boolean operations, the union of these different elements is essential. For the octree with depth *ϐ De*, the octree root cell is initialized as the bounding box of the offset surface.

To optimize memory for this model size, we further discretize these bounding boxes into smaller voxels that are merged into a unified surface later. The voxel layer is divided into overlapping tiles to ensure a tight surface. For a maximum octree refinement ($\varkappa$) and a grid of tiles (*grid*), the voxel grid is ${((2 \varkappa -1) grid + 1)}^{3}$. To eliminate invalid self-intersecting geometries in tight urban canyons Fig. 3a, we use a filtration condition where the invalid surface polygons are removed when they do not have a neighbor polygon with ${\delta }_{o}$ ≤ minimum offset distance. Remaining polygons are processed utilizing a modified Dual Contouring Algorithm^71,72^. The modified method is adjusted for model processing to optimize computational power and eliminate noise. In Rhinoceros modelling the obstacle boundary $O$ for each polygon $e$, the minimum normal vector to the tangent polygon plane $\overline{\overline{O}}$, center projection $\widehat{\upsilon }$, and the surface sample projection ${\widehat{\upsilon }}{\prime}$ are given by:

1 $${\overline{\overline{O}}}^{T}={\overline{\overline{O}}}^{T}\boldsymbol {w}_{min}+{\delta }_{o} - {\delta }_{min}$$

2 $${\widehat{\upsilon}}{\prime}=\widehat{\upsilon}+ \overline{\overline{O}} ({\overline{\overline{O}}}^{T}({w}_{min}- \widehat{\upsilon }) + {\delta }_{o} - {\delta }_{min})$$

where ${\delta }_{min}$ is the minimum offset distance; ${w}_{min}$ is the minimum offset distance point on polygon.

To guarantee the generated cells lie on the surface, a smoothing mesh function is utilized. For the final generated offset mesh *M*, a relaxation force pulls every vertex ${\upsilon }_{h}$ in the mesh vertices towards ${\upsilon }{\prime}$ while offset force pulls $\upsilon$ towards ${\upsilon }^{{\prime}{\prime}}$ as follows:

3 $${\upsilon }^{{{\prime}}}= \frac{1}{w} \sum_{h}{\upsilon }_{h}$$

4 $${\upsilon }^{{\prime}{\prime}}=\widehat{b}\boldsymbol +\boldsymbol {\delta }_{o} \frac{\upsilon - \widehat{b}}{\Vert \upsilon - \widehat{b}\Vert }$$

where $\widehat{b}$ is the base point on mesh $M$ with the minimum distance to ${\upsilon }_{h}$. This mesh relaxation techniques allow the inclusion of tight urban geometries, which could significantly impact the airspace capacity. Furthermore, it allows a better applicability for other keep-in geofences that we are not using in the study such as the shape method by Edelsbrunner et al.^73^.

**Figure 4:**
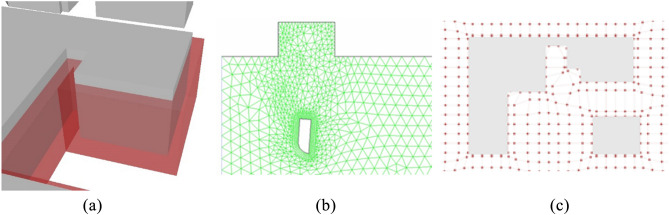
(**a**) Self-intersecting urban mass, Grey (original geometry), Red (offset geometry). (**b**) CFD meshing^75^. (**c**) Dynamic meshing illustrated on urban mass after smoothing.

The smoothed mesh helps with the adoption of a novel dynamic meshing technique similar to CFD in buildings simulations^74^. The dynamic mesh accommodates and changes according to the model space, in self-intersecting geometries Fig. 4a or around obstacles, the mesh gets stricter (i.e., the spacing between graph vertices gets smaller) and vice versa in wider or obstacle-free areas where the mesh spacing gets wider as illustrated in Fig. 4b,c. This cartesian meshing will be explained in “Trip generation, cartesian routing, and UAV energy consumption” for graph-based solvers.

To proceed with airspace planning, we create a virtual box with the bottom as the 3D model ground surface, side boundaries taken from the simulated city patch and the top is constructed at the maximum flight altitude (*β*_*max*_) given from the simulated policy. A Boolean subtraction process subtracts the entire 3D model with an offset value of the minimum horizontal distance from property as the *δ*_*o*_ offset value from the airspace boundary virtual box. The resultant volume (*Ꞙ*) is the UAV motion viable airspace.

To keep the Digital-Twin updated, loop 1 is performed within a predetermined time-step to input the updated LiDAR data with any significant changes that might cause disruption. Data is processed in (1–8); thereafter, the airspace discretization model is updated. A queen UAV is expected to update in the defined time step. In this study, we utilize a combination of variance estimation model-driven and point cloud-based Iterative Closest Point (ICP) methods to align the geometry of two roughly pre-registered, partially overlapping, rigid, noisy 3D point sets^76^. The code is written in Python.

Given the stored city model mesh set *M* and the new LiDAR dataset M*, for each point ${\upsilon }_{h}^{*} \in$ ∂M*, we allocate the closest complementing point(s) ${\upsilon }_{h}$ in *M*. Consequently, we compute the incremental transformation using a weighted least-squares function given by^77^:

5 $$min_{{\left( {Q,\vec{T}} \right)}} \sum\nolimits_{h} {\omega _{h} } \left\| {M_{h} - \left( {QM^{*} _{h} + \vec{T}} \right)} \right\|^{2}$$

where *Q* is rotation matrix, $\overrightarrow{T}$ is the translation vector. The weighing variable ${\omega }_{h}$ is set to zero if the Euclidean distance (ED) between ${\upsilon }_{h}^{*}$ and ${\upsilon }_{h}$ defined as (*d*_*h*_ $\triangleq$ *d* (${\upsilon }_{h}^{*}$, ${\upsilon }_{h}$)) is larger than the maximum tolerance threshold *δ*_*max*_ set to 1 m in this study. This determines the motion in existing elements of the model such as limited movement urban elements (trees, scaffolds, and construction equipment). However, new elements in the LiDAR data that falls within the boundary of (*Ꞙ*) are added as identified obstacles *O* undergoing the process in (1–8) to be integrated with the new mesh set *M*.

## Airspace discretization model description

In this section, the proposed airspace discretization method is formally explained. A brief explanation of the UAV flight trajectory dynamics is presented in “Dynamic trajectory properties” The novel airspace discretization model morphology is discussed in “Airspace discretization morphology”. The proposed keep-in geofence and geometrical disruption of UAV flight trajectories (*Skyroutes algorithm*) are discussed in “Robust Skyroutes algorithm”.

### Dynamic trajectory properties

**Figure 5:**
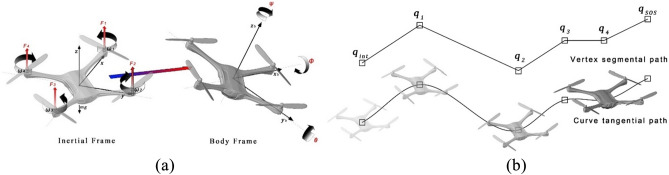
Quadcopter motion dynamics. Source: Authors created by Rhinoceros 3D, version 7, rhino3d.com. (**a**) Inertial and body frames, and Euler angles φ, θ and ψ. (**b**) Vertex transition vs curve transition path.

In this study, we utilize two different airspace discretization methods, namely, segment-based and cartesian-based. While segmental discretization includes a path geometrical optimality component, cartesian-based paths are, by default, generated as a set of straight-line segmented polyline paths. The complexity of the geometry depends on the mission initiation and destination locations, flight policy, and the characteristics of the obstacles to be evaded. Since UAVs propagate along a continuous trajectory, a hard-angled segmented flight path is not feasible or may lead to overshooting from the keep-in geofence. Similarly, the integration between both types of generated paths in a single flight plan requires a viable geometric transition Fig. 5a.

In the literature, this problem was tackled by Bézier curves to reform the generated flight path^78^. However, common generating algorithms of Bézier curves can tend to be computationally inefficient^79^. In this study, interpolated fit-point ‘cubic’ splines are adopted for computational efficiency. The method is based on B-spline interpolation function and UAV motion equations. A B-spline with fit points transitions from cartesian point cloud reference is utilized to reform vertex to curve transition (Fig. 5b). For the UAV, the generated path is a B-spline rather than a set of straight-line segments polyline. Although for cartesian discretization, most optimal trajectory generation algorithms (Table 2) adopt their individual methodologies to generate the most energy efficient trajectories. However, this base B-spline method is needed as the shortest path for optimization in some methods. The curve equation for path correction is given by:

6 $$\overline{\text{C} }(q)=\sum_{\widehat{i}=0}^{\widehat{k}} {N}_{\widehat{i},deg}(q){\overline{\mathbf{P}} }_{\widehat{i}}$$

where $\widehat{k}$ is the number of vertices along the trajectory; *N* is the matrix of B-spline basis functions for vertices ${q}_{\widehat{i}}$ to ${q}_{\widehat{i}+1}$; the degree of curvature is determined by ($deg$) based on the UAV kinematics, the detailed iterative process is outside the scope of this study; and ${\overline{\mathbf{P}} }_{\widehat{i}}$ is the matrix of curvature degrees for vertices ${q}_{\widehat{i}}$ to ${q}_{\widehat{i}+1}.$

### Airspace discretization morphology

The four different airspace discretization morphologies are discussed in the literature and summed up in “Urban airspace planning”, Fig. 1. In Elsayed and Mohamed^22,80–82^, the impact of airspace regulations and flight path geometry/trajectory on the energy consumption and GHG emissions was illustrated, however, the study results showed the challenge of failed missions. In this study, we overcome the mission failure and inviable trajectories by proposing a novel logistic dynamic discretization morphology that combines the advantages from each discretization method and eliminates the disadvantages.

**Figure 6:**
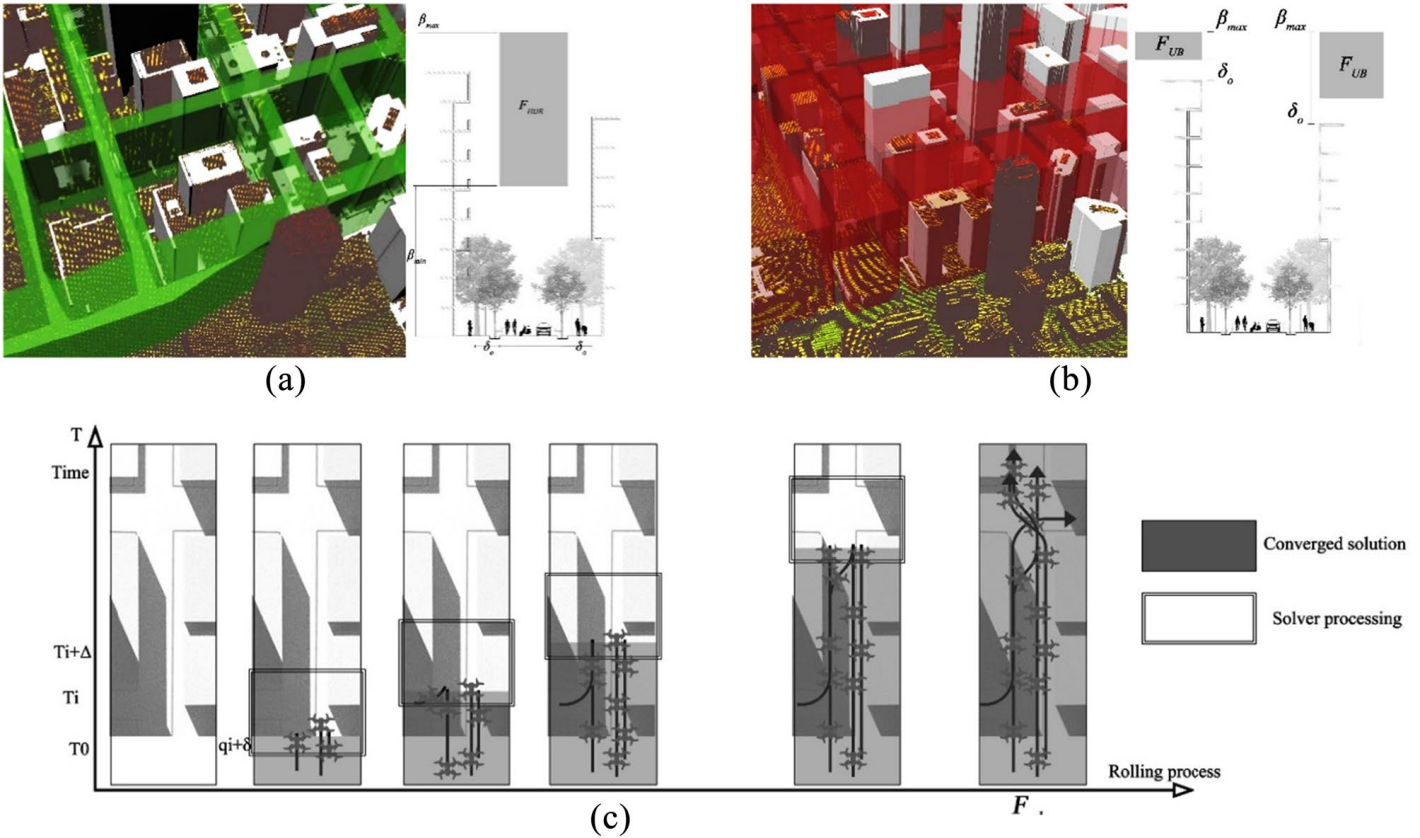
Proposed discretization morphology. (**a**) F_HDR_ High density routes airspace. (**b**) F_UB_ Urban block (UB) airspace. (**c**) Rolling horizon airspace discretization framework.

Starting with city obstacle mesh M, the city’s viable airspace can be divided into two volumetric sets *F*_*HDR*_ and *F*_*UB*_. *F*_*HDR*_ can be defined as High density routes (HDR) airspace where all missions connecting different city blocks will have to navigate to comply with the flight regulations. This is illustrated in Fig. 6a, and it is the volume mostly aligning with the city major road network starting from minimum flight altitude (*β*_*min*_) up to maximum flight altitude (*β*_*max*_). This volume is obstacle free with a minimum clearance distance of (*δ*_*o*_) from the nearest obstacle. *F*_*HDR*_ is further discretized in “Robust Skyroutes algorithm” into a hybrid model between Layers, zones, and tubes.

In comparison, *F*_*UB*_ can be defined as Urban block (UB) airspace illustrated in Fig. 6b. It is the air volume above buildings aligning with city urban blocks specifically between major roadways. The airspace starts from a minimum clearance distance of (*δ*_*o*_) from the obstacles (buildings and others). Similar to *F*_*HDR*_, it extends up to the maximum flight altitude (*β*_*max*_). Origins and destinations without major road access will only have access to the airspace through the air volume above these blocks to access the *F*_*HDR*_ network.

Given the size of the discretized model and complexity of the details, especially the number of geometrical intersecting features, computational complexity grows quickly with the number of obstacle patches ∂*O* and the timestep *t*. The greatest challenge of all is the enforcement of the geofence constraints simultaneously with the UAV constraints, to ensure safe operations. These constraints also become increasingly difficult to process as the 3D urban model consumes the memory allocation, and the UAV mission demand increases, thereby creating more potential conflicts. To reduce the computational complexity and maximize memory usage, we adopt a commonly used strategy to dissect the problem through a rolling horizon framework (Fig. 6c). Rolling horizon has been applied to solve a variety of time-dependent optimization problems in aerial transport such as aircraft scheduling^83^.

Instead of discretizing the entire city obstacle mesh *M*, we divide the set into a series of subproblems, each defined by an initial coordinates *q*_*o*_ ∈ *Ꞙ* and a rolling processing window {*q*_*i*_, *q*_*i*+Δ_} where [Δ $\ll$ *F*_*edge*_]. We ensure overlapping in the solution by reiterating the last section {*q*_*i*_ , *q*_*i*+ *η*_} where [*η* $\ll$ Δ] after a subproblem converges. This overlap reduces the possibility of redundant or invalid solutions and guarantees accounting for all obstacles. The rolling horizon method is illustrated in Fig. 6c, where solid rectangles represent the ongoing discretization process at current timestep, and grey zones highlight the converged solutions saved in the memory.

Given the *F*_*HDR*_ cross section at *J*_*o*_ such as in Fig. 6a,b, we construct a polygonal vertical surface and contour it horizontally and vertically to dimensions $\widehat{A}$ and $\widehat{B}$ respectively, where {$\widehat{A}$ ≤ (Street width) − (2 × *δ*_*o*_)} and {$\widehat{B}$ ≤ *β*_*max*_—*β*_*min*_}. ($\widehat{A}$) will determine the maximum allocation of UAV lanes horizontally, and ($\widehat{B}$) will determine the maximum allocation of lanes vertically. Equations (11–13) for contouring are illustrated in the appendix. We can utilize the maximum area of each inscribed polygon for UAV lanes. This ensures maximum capacity and avoids the formation of bottle necks, which will require further lane traffic management and will decrease the traveling speed and energy utilization.

**Figure 7:**
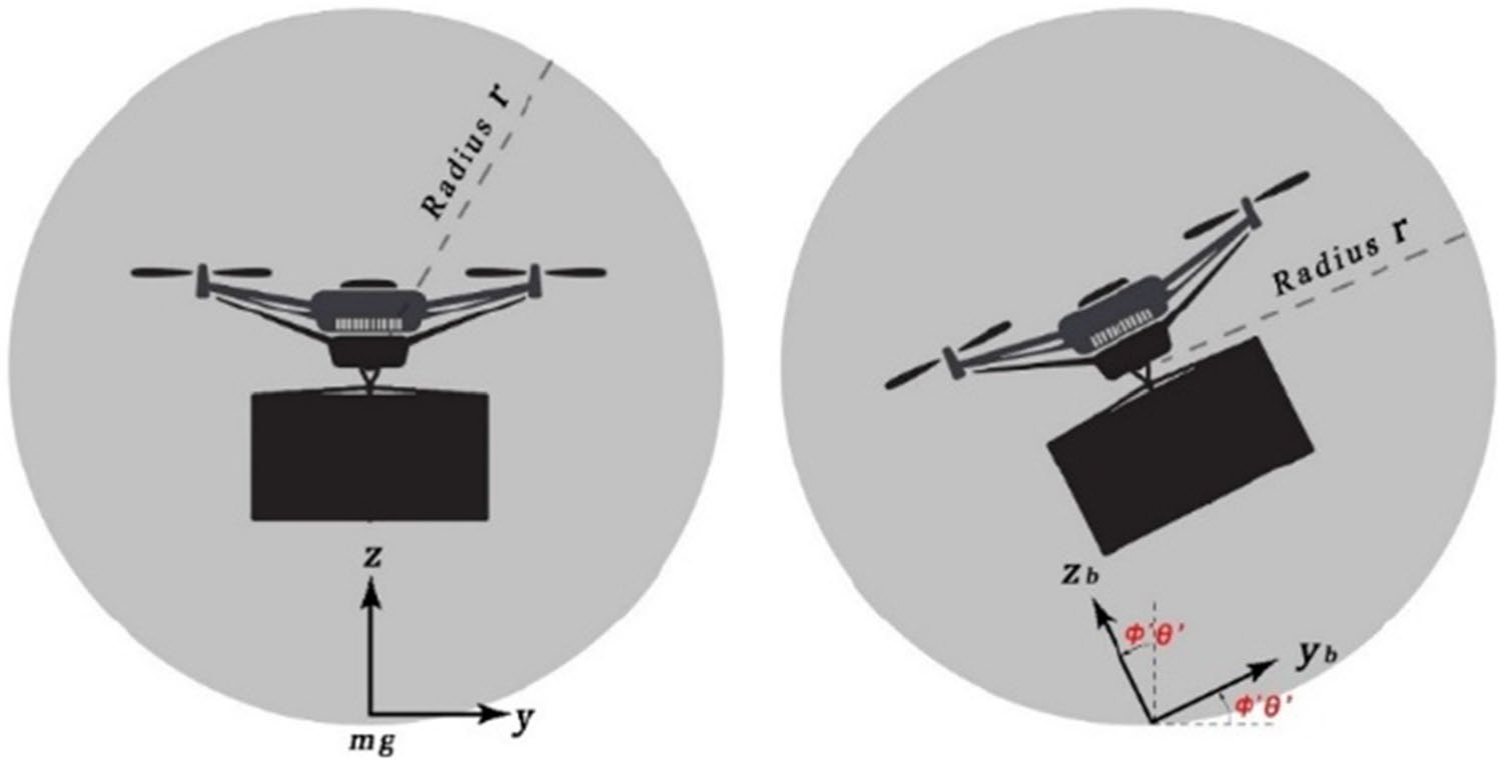
Payload motion within circular keep-in geofence.

Whether the payload is confined in the UAV frame or suspended by a wire, during the UAV motion around the pitch, roll, and yaw angles, the payload will swing with motion, especially with aggressive maneuvers. It is crucial to reduce the payload oscillation to avoid damage and guarantee safe operation. Hence, we design the UAV keep-in geofence to account for the payload motion as illustrated in Fig. 7.

In cross section, the UAV lane can be considered a circle with radius *r*. Assuming an *F*_*HDR*_ airspace volume with dimensions $\widehat{A}$ and $\widehat{B}$ starting from cross section *J*_*o*_ to *J*_*k*_, we can consider the horizontal lanes as lofted cylinders. Hence, a typical cylinder packing problem is used. In 2D, this problem is equivalent to the circle packing problem where the aim is to maximize the airspace capacity of lanes (circles) while maintaining the minimum radius *r* such as in Birgin et al.^84^. To maximize the lane airspace capacity, given $\gamma$ lanes of radius *r* and a polygonal *F*_*HDR*_ airspace, we utilize a nonlinear optimization model to solve the problem as follows:

7 $$\text{min}{\sum }_{\widetilde{i}=1}^{\gamma }{\sum }_{\widetilde{j}>\widetilde{i}}^{\gamma }{\text{max}\left(\text{0,4}{r}^{2}-{d}_{\widetilde{i}\widetilde{j}}^{2}\right)}^{2}$$

8 $$\begin{gathered} s.t. r \le x_{{\tilde{i}}} \le \hat{A} - r,\quad \tilde{i} = 1, \ldots ,\gamma , \hfill \\ r \le y_{{\tilde{i}}} \le \hat{B} - r,\quad \tilde{i} = 1, \ldots ,\gamma . \hfill \\ \end{gathered}$$

By equating the objective function to zero, if the lanes fit in the cross section, the solver terminates and inscribes the circles.

Given the centers of circles in (11–15), UAVs start at *j*; and *j*_*k*_ is the destination. We can extrapolate the keep-in geofence volumetric tubes with vector flight velocity $\widetilde{v}$. Where *q*_=_ (x, y, z) is defined as the initial location coordinates for UAV aligned with the center of circular keep-in geofence number ($\widetilde{i}$) within the cartesian referencing system. The orthogonal geofence grid of UAV pathways (lanes) are modeled by scaling up Peng et al. Algorithm^85^:

9 $$\widetilde{v}(q\text{) = }{\left(\frac{\overline{\overline{v}}\left(x-{x}_{k}\right)}{\sqrt{{\left(x-{x}_{k}\right)}^{2}+{\left(y-{y}_{k}\right)}^{2}+{\left(z-{z}_{k}\right)}^{2}}}, \frac{\overline{\overline{v}}\left(y-{y}_{k}\right)}{\sqrt{{\left(x-{x}_{k}\right)}^{2}+{\left(y-{y}_{k}\right)}^{2}+{\left(z-{z}_{k}\right)}^{2}}}, \frac{\overline{\overline{v}}\left(z-{z}_{k}\right)}{\sqrt{{\left(x-{x}_{k}\right)}^{2}+{\left(y-{y}_{k}\right)}^{2}+{\left(z-{z}_{k}\right)}^{2}}}\right)}^{Tr}$$

At flight velocities over 3 m/s, translational lift increases the power efficiency significantly. While the speed profile will vary based on the path geometry and the status of the UAV (loaded or unloaded). To achieve the best energy efficiency, constant $\overline{\overline{v}}$ speeds are maintained above 10 m/s and below 20 m/s to maintain the viable route while capitalizing battery utilization. It is worth noting that UAV energy limitations will still apply based on battery capacity.

**Figure 8:**
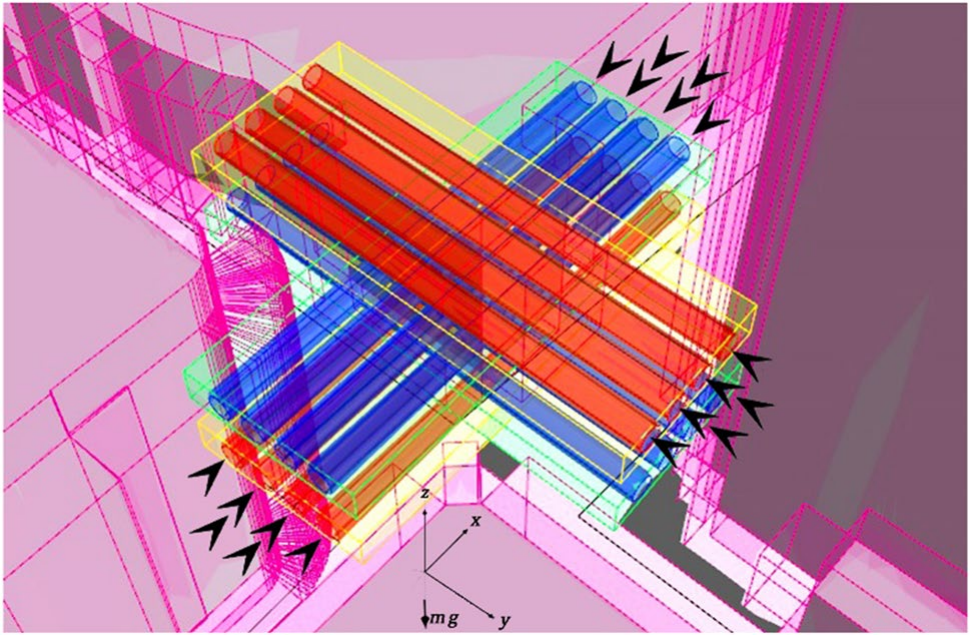
Proposed hybrid layered, zonal, and tubed discretization.

Figure 8 shows the proposed morphology combining layered, zonal, and tubed discretization. For each flight bearing (eastbound, westbound, northbound, and southbound) the lanes are superimposed (layered) for two objectives; (1) avoid potential intersection, (2) allow empty space above and below the keep-in geofence for lane merging on left and right turns. The layers are shown in yellow and green depending on the flight direction. Furthermore, the tubes (circular lane keep-in geofence) are represented in blue and red depending on the vector of flight direction. The arrows in Fig. 8. represent the heading of vector lane velocity $\widetilde{v}$ which is organized to allow slower $\overline{\overline{v}}$ speeds on the rightmost and leftmost lanes and highest $\overline{\overline{v}}$ speeds towards the middle. The zones represented in magenta are the individual property buffer acting as ‘ramps’ for UAVs taking off/landing from the street-level or balconies/ terraces. These zones are NFZs except for authorized UAVs.

While the proposed framework can function at this level efficiently, “Robust Skyroutes algorithm” illustrates the geometrical modification based on UAV kinematics, which is essential with each digital twin model update and in case of disruption or complex geometrical street grids with obstacle protrusions.

### Robust Skyroutes algorithm

To model the lane disruption, we develop based on disturbed flow to generate sky lanes by scaling up the Peng et al. VF-based novel algorithm^85^ and the interfered fluid dynamical system^72^, we describe obstacles as attractive fields through a function. Obstacles in mesh *M* registered after the smoothing process in (9) can be expressed here as a finite set of welded simplified volumes in cartesian planning space (*x*, *y*, *z*), each with a geometrical center at *q*_*obs* =_ (*x*_*obs*_,* y*_*obs*_,* z*_*obs*_), and axial dimensions $\left({x}_{\delta},{y}_{\delta },{z}_{\delta }\right)$. The obstacle function becomes:

10 $$F\left(q\right) = {\left(\frac{x -{x}_{\text{obs}}}{{x}_{\delta }}\right)}^{2}+ {\left(\frac{y -{y}_{\text{obs}}}{{y}_{\delta }}\right)}^{2}+ {\left(\frac{z -{z}_{\text{obs}}}{{z}_{\delta }}\right)}^{2}$$

where *q*_=_ (*x*, *y*, *z*) is defined as the UAV inertial frame location coordinates within the point cloud referencing system. While the proposed method is based on the artificial potential field (APF) method by^86^ in modeling the disruption, however, the proposed method is more robust with a single solution rather than a local optimum. The disruption function D(*q*) and the modified vector flight velocity $\overline{v }$ at any timestep can be determined utilizing the effective matrix of obstacles (${B}_{obs}$) in obstacle boundary set ($u$*; where* $u$ *∈* ∂*O*) impacting the UAV lanes as follows:

11 $$\begin{aligned}\text{D}({\text{q}}) & = {B}_{obs }-\frac{ {\left[\frac{\partial F\left(q\right)}{\partial x},\frac{\partial F\left(q\right)}{\partial y},\frac{\partial F\left(q\right)}{\partial z}\right]}^{\text{Tr}}. \left[\frac{\partial F\left(q\right)}{\partial x},\frac{\partial F\left(q\right)}{\partial y},\frac{\partial F\left(q\right)}{\partial z}\right]}{{\left|\text{F}\left(q\right)\right|}^{{{\delta }_{V }\text{exp }\left(1-\frac{1}{\sqrt{{\left({x}_{u}-x\right)}^{2}+{\left({y}_{u}-y\right)}^{2}+{\left({z}_{u}-z\right)}^{2}} . \sqrt{{\left(x-{x}_{k}\right)}^{2}+{\left(y-{y}_{k}\right)}^{2}+{\left(z-{z}_{k}\right)}^{2}}}\right)}^{-1}}. \left[\frac{\partial F\left(q\right)}{\partial x},\frac{\partial F\left(q\right)}{\partial y},\frac{\partial F\left(q\right)}{\partial z}\right]. {\left[\frac{\partial F\left(q\right)}{\partial x},\frac{\partial F\left(q\right)}{\partial y},\frac{\partial F\left(q\right)}{\partial z}\right]}^{\text{Tr}}}\\ &\quad+ \frac{{\left[\frac{\partial F\left(q\right)}{\partial y},-\frac{\partial F\left(q\right)}{\partial x},0\right]}^{\text{Tr}}. \left[\frac{\partial F\left(q\right)}{\partial x},\frac{\partial F\left(q\right)}{\partial y},\frac{\partial F\left(q\right)}{\partial z}\right] }{{\left|F\left(q\right)\right|}^{{{\delta }_{H }\text{exp }\left(1-\frac{1}{\sqrt{{\left({x}_{u}-x\right)}^{2}+{\left({y}_{u}-y\right)}^{2}+{\left({z}_{u}-z\right)}^{2}} . \sqrt{{\left(x-{x}_{k}\right)}^{2}+{\left(y-{y}_{k}\right)}^{2}+{\left(z-{z}_{k}\right)}^{2}}}\right)}^{-1}} . \Vert {\left[\frac{\partial F\left(q\right)}{\partial y},-\frac{\partial F\left(q\right)}{\partial x},0\right]}^{\text{Tr}}\Vert . \Vert {\left[\frac{\partial F\left(q\right)}{\partial x},\frac{\partial F\left(q\right)}{\partial y},\frac{\partial F\left(q\right)}{\partial z}\right]}^{\text{Tr}}\Vert }\end{aligned}$$

12 $$\overline{v}(q) = \widetilde{v}(q)\text{D}({\text{q}})$$

where the lane trajectory angle to the obstacle is denoted by *δ*_*V*_ for the vertical policy parameter and *δ*_*H*_ the horizontal policy parameter.

#### Lemma 1

* Assuming the perpendicular and tangential vectors form a right angle, and* ${p (q) }^{Tr}. \overline{v }(q) = 0$.* It indicates that the trajectory lanes can avoid obstacles [B*_obs_*] within legally allowed tolerances* ${[\delta }_{min}]$.

#### Lemma 2

$\overline{v }(q) . {\widetilde{v}(q)}^{Tr} \ge 0$, *which indicates that the trajectory can successfully reach the segment destination [J*_*k*_].

#### Lemma 3

$\overline{v}\propto {\delta }_{V}$ *It indicates that the magnitude of the repulsive and tangential trajectory velocity is directly proportional to the lane proximity horizontal and vertical policy parameters. i.e., following the edge of the boundary of effective matrix of obstacles* *B*_*obs*_ *precisely is inversely proportional to the proximity of the lane to the avoided obstacles.*

#### Theorem 1

*If Lemma 1 is satisfied, Lemma 2 is satisfied, and Lemma 3 is satisfied for any obstacle set in mesh* *M*, *we can guarantee the feasibility of UAV traffic lanes and trajectories.*

#### L.1. Proof

Suppose the perpendicular vector the UAV trajectory is *p* (*q*); and the tangential vector to the UAV trajectory *t* (*q*) at point ${q}_{i}$ on the surface of a single obstacle within mesh *M* are perpendicular the following from Eq. (17) and (18) stands true:

13 $$F\left(q\right) =1$$

14 $$\text{D} ({\text{q}}) = {B}_{\text{obs }}-\frac{ {\left[\frac{\partial F\left(q\right)}{\partial x},\frac{\partial F\left(q\right)}{\partial y},\frac{\partial F\left(q\right)}{\partial z}\right]}^{\text{Tr}}. \left[\frac{\partial F\left(q\right)}{\partial x},\frac{\partial F\left(q\right)}{\partial y},\frac{\partial F\left(q\right)}{\partial z}\right]}{\left[\frac{\partial F\left(q\right)}{\partial x},\frac{\partial F\left(q\right)}{\partial y},\frac{\partial F\left(q\right)}{\partial z}\right]. {\left[\frac{\partial F\left(q\right)}{\partial x},\frac{\partial F\left(q\right)}{\partial y},\frac{\partial F\left(q\right)}{\partial z}\right]}^{\text{Tr}}} + \frac{{\left[\frac{\partial F\left(q\right)}{\partial y},-\frac{\partial F\left(q\right)}{\partial x},0\right]}^{\text{Tr}}. \left[\frac{\partial F\left(q\right)}{\partial x},\frac{\partial F\left(q\right)}{\partial y},\frac{\partial F\left(q\right)}{\partial z}\right] }{ \Vert {\left[\frac{\partial F\left(q\right)}{\partial y},-\frac{\partial F\left(q\right)}{\partial x},0\right]}^{\text{Tr}}\Vert . \Vert {\left[\frac{\partial F\left(q\right)}{\partial x},\frac{\partial F\left(q\right)}{\partial y},\frac{\partial F\left(q\right)}{\partial z}\right]}^{\text{Tr}}\Vert }$$

15 $$\text{Therefore}, {p (q) }^{Tr}. \overline{v }(q) ={ p (q) }^{Tr}. {\widetilde{v} (q). }\text{D} ({\text{q}})= {\widetilde{v}(q)}.\left({{\text{p}}({\text{q}})}^{Tr}-{{\text{p}}({\text{q}})}^{Tr}+\frac{{{\text{p}}({\text{q}})}^{Tr}. {\text{t}}({\text{q}}) .{ \, {\text{p}}({\text{q}})}^{Tr}}{ \Vert {\text{t}}({\text{q}})\Vert . \Vert {\text{p}}({\text{q}})\Vert } \right)=0$$

**Figure 9:**
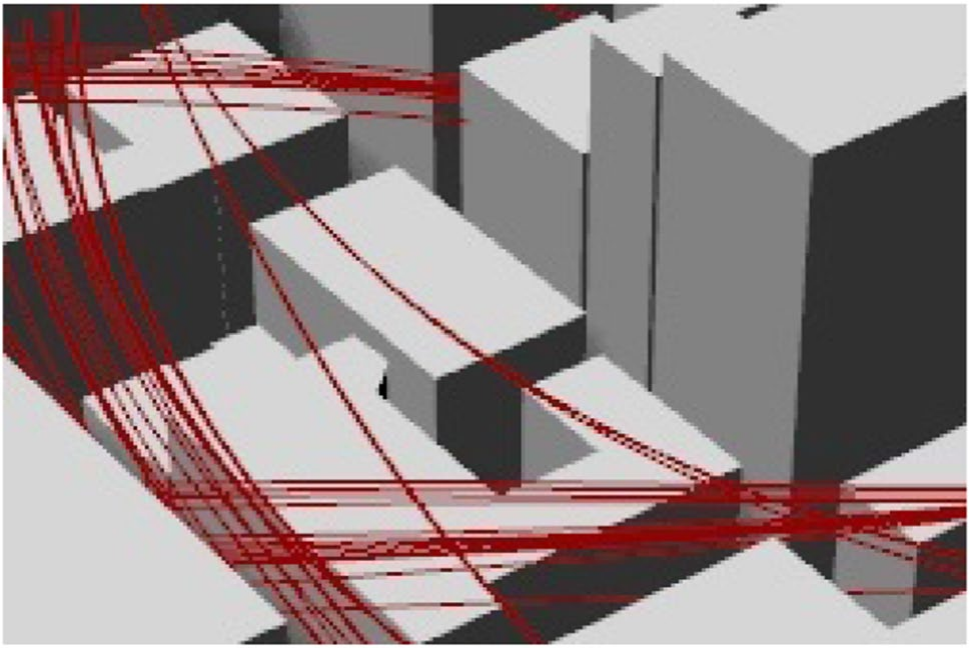
Lemma 1trajectory avoiding concave obstacle trap areas [B_obs_] within legally allowed tolerances.

From (22) we can deduce that with the absence of a value for the perpendicular component, the UAV trajectory using the artificial potential field generated path will not intersect with the obstacle mesh. Figure 9 shows the generated trajectory avoiding a concave tight obstacle trap area.

#### L.2. Proof

Given the mission’s distance between takeoff and landing (*J* and *J*_*k*_) is relatively short, theoretically $\overline{v }{ \approx }\widetilde{v}$, applying these yields

$$\overline{v } (q) . {\widetilde{v}(q)}^{Tr} = {\widetilde{v} (q)}^{Tr}. \widetilde{v} (q). \text{D }({\text{q}})$$

$$= {\Vert \widetilde{v} (q)\Vert }^{2}$$

$$\left(1-\frac{{\left(\text{cos}\alpha \right)}^{2}}{{\left|\text{F}\left(q\right)\right|}^{{{\delta }_{V }\text{exp }\left(1-\frac{1}{\sqrt{{\left({x}_{u}-x\right)}^{2}+{\left({y}_{u}-y\right)}^{2}+{\left({z}_{u}-z\right)}^{2}} . \sqrt{{\left(x-{x}_{k}\right)}^{2}+{\left(y-{y}_{k}\right)}^{2}+{\left(z-{z}_{k}\right)}^{2}}}\right)}^{-1}}}\right)+$$

16 $$\frac{{\left[\frac{\partial F\left(q\right)}{\partial y},-\frac{\partial F\left(q\right)}{\partial x},0\right]}^{\text{Tr}}. \left[\frac{\partial F\left(q\right)}{\partial x},\frac{\partial F\left(q\right)}{\partial y},\frac{\partial F\left(q\right)}{\partial z}\right] }{ {\left|F\left(q\right)\right|}^{{{\delta }_{H }\text{exp }\left(1-\frac{1}{\sqrt{{\left({x}_{u}-x\right)}^{2}+{\left({y}_{u}-y\right)}^{2}+{\left({z}_{u}-z\right)}^{2}} . \sqrt{{\left(x-{x}_{k}\right)}^{2}+{\left(y-{y}_{k}\right)}^{2}+{\left(z-{z}_{k}\right)}^{2}}}\right)}^{-1}}\Vert {\left[\frac{\partial F\left(q\right)}{\partial y},-\frac{\partial F\left(q\right)}{\partial x},0\right]}^{\text{Tr}}\Vert . \Vert {\left[\frac{\partial F\left(q\right)}{\partial x},\frac{\partial F\left(q\right)}{\partial y},\frac{\partial F\left(q\right)}{\partial z}\right]}^{\text{Tr}}\Vert }$$

Taking *α* is the deviation angle between the vector flight velocity *v*_*l*_ and the obstacle perpendicular vector to the UAV trajectory. While ${\text{F}} ({\text{q}}) \ge 1$ and ${(\text{cos}\alpha )}^{2}\le 1$ we can deduct:

17 $$\frac{1- \, {\left({cos}\alpha \right)}^{2}}{{\left|F\left(q\right)\right|}^{{{\delta }_{V }\text{exp }\left(1-\frac{1}{\sqrt{{\left({x}_{u}-x\right)}^{2}+{\left({y}_{u}-y\right)}^{2}+{\left({z}_{u}-z\right)}^{2}} . \sqrt{{\left(x-{x}_{k}\right)}^{2}+{\left(y-{y}_{k}\right)}^{2}+{\left(z-{z}_{k}\right)}^{2}}}\right)}^{-1}}}\ge 0;$$

18 $${\left[\frac{\partial F\left(q\right)}{\partial y},-\frac{\partial F\left(q\right)}{\partial x},0\right]}^{\text{Tr}}. \left[\frac{\partial F\left(q\right)}{\partial x},\frac{\partial F\left(q\right)}{\partial y},\frac{\partial F\left(q\right)}{\partial z}\right]\ge 0$$

19 $$\therefore \overline{v }(q) .{\widetilde{v}(q)}^{Tr} \ge 0$$

From (26) we can deduce that the UAV trajectory will successfully reach the segment destination [*J*_*k*_].

**L.3. Proof.** The vector flight velocity $\overline{v } \,$ at any timestep which was explained in (19) can be rewritten as:

$$\overline{v }(q) = \widetilde{v}(q)\text{D}({\text{q}})$$

20 $$\bar{v}(q) = \widetilde{v} (q) - \frac{{p (q) ^{{Tr}} . \widetilde{v} (q)}}{{\left| {F\left( q \right)} \right|^{{\delta _{V} (q)^{{ - 1}} }} .p (q) ^{{Tr}} .p (q)}}p (q) + \frac{{p (q) ^{{Tr}} . \tilde{v} (q)}}{{\left| {F\left( q \right)} \right|^{{\delta _{H} (q)^{{ - 1}} }} .\left\| {t (q)} \right\|.\left\| {p (q)} \right\|}}t (q)$$

Similar to the disruption function in (20) three terms are described; $\widetilde{v}(q)$ can be explained as the attractive velocity given by the maximum UAV allowed speed by the applicable civil flight regulation policy; second term [$\frac{{{\text{p}}({\text{q}})}^{Tr}. \widetilde{v}(q)}{{\left|F\left(q\right)\right|}^{{{\delta }_{V }(q)}^{-1}}. {{\text{p}}({\text{q}})}^{Tr}. {\text{p}}({\text{q}})} {\text{p}}({\text{q}})$] is the APF repulsive velocity; [$\frac{{{\text{p}}({\text{q}})}^{Tr}. \widetilde{v}(q)}{{\left|F\left(q\right)\right|}^{{{\delta }_{H }(q)}^{-1}}. \Vert {\text{t}}({\text{q}})\Vert . \Vert {\text{p}}({\text{q}})\Vert } {\text{t}}({\text{q}})$] is the APF tangential velocity.

**Figure 10:**
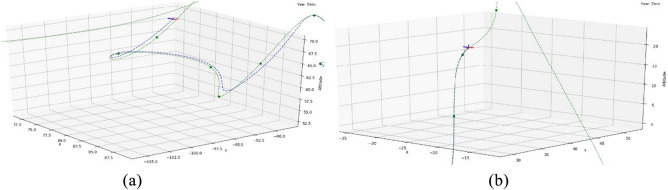
(**a**) UAV failing to maintain lane trajectory. (**b**) UAV following lane trajectory.

The concept behind controlling the trajectory is to avoid off-shooting and reduce the risk factor (ξ), this is defined as the possibility of UAV derailing from the designated lane or trajectory, hence risking potential collision or traffic disruption. Figure 10 shows a UAV failing to maintain trajectory due to the path infeasibility or kinematic incompatibility.

**Figure 11:**
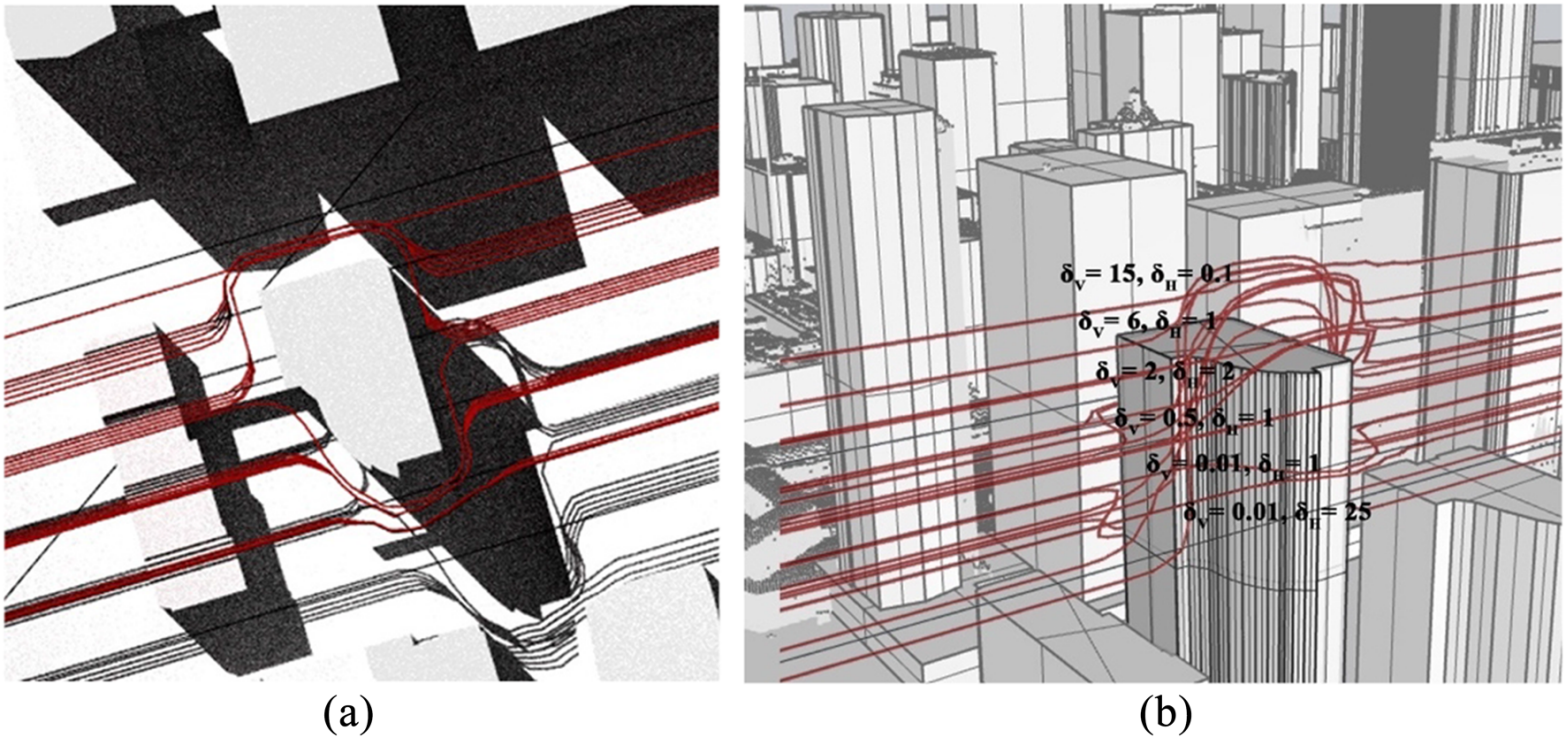
(**a**) UAV lane trajectory options. (**b**) Different combinations of ${\delta }_{V}$ and ${\delta }_{H}$.

To achieve the maximum speed on a feasible path while ensuring a consistent keep-in geofence, either the speed is reduced leading to time/energy consumption inefficiencies^80^, or the trajectory is modified. Different combinations of ${\delta }_{V } and {\delta }_{H}$ are tested such as in Fig. 11. As illustrated, the produced trajectory shows that the magnitude of the repulsive and tangential trajectory velocity is directly proportional to the lane proximity horizontal and vertical policy parameters.

Based on the numerical formula, an algorithm is developed for trajectory propagation dynamics. This algorithm is capable of solving both the discretization and trajectory planning problems in a three-dimensional environment. The algorithm has a unique two-step procedure. The process of the algorithm can be explained as follows:

- Initialization and loop 1: the algorithm starts by setting up the environment, including the grid, obstacles, origins, destination points (O–D), and other parameters necessary for trajectory planning. This stage aims at adjusting a 3D solid mesh for obstacles using freeform object reconstruction, tangent calculation, and offset processing to generate the mesh of obstacles with the keep-out geofence based on the updated digital twin and flight policies.
- Main loop: the core of the algorithm involves iterating through potential paths and dynamically adjusting them based on the evolving environment and obstacle positions by modifying theoretic trajectories (‘sky routes’ at policy-allowed elevations) with expected flight velocity $\overline{v }(q)$ at every time step, which is adjusted according to the presence of obstacles. This is achieved using a reformation disruption matrix; vertical and horizontal tangential deformations are imposed on the matrix “D (q)” and considering the UAV’s maximum allowed speed, the repulsive velocity from obstacles, and the tangential velocity to navigate around obstacles. This ensures that the UAV’s path avoids collisions and maintains a safe and viable trajectory.

The algorithm in pseudocode is presented as follows:

**Figure:**
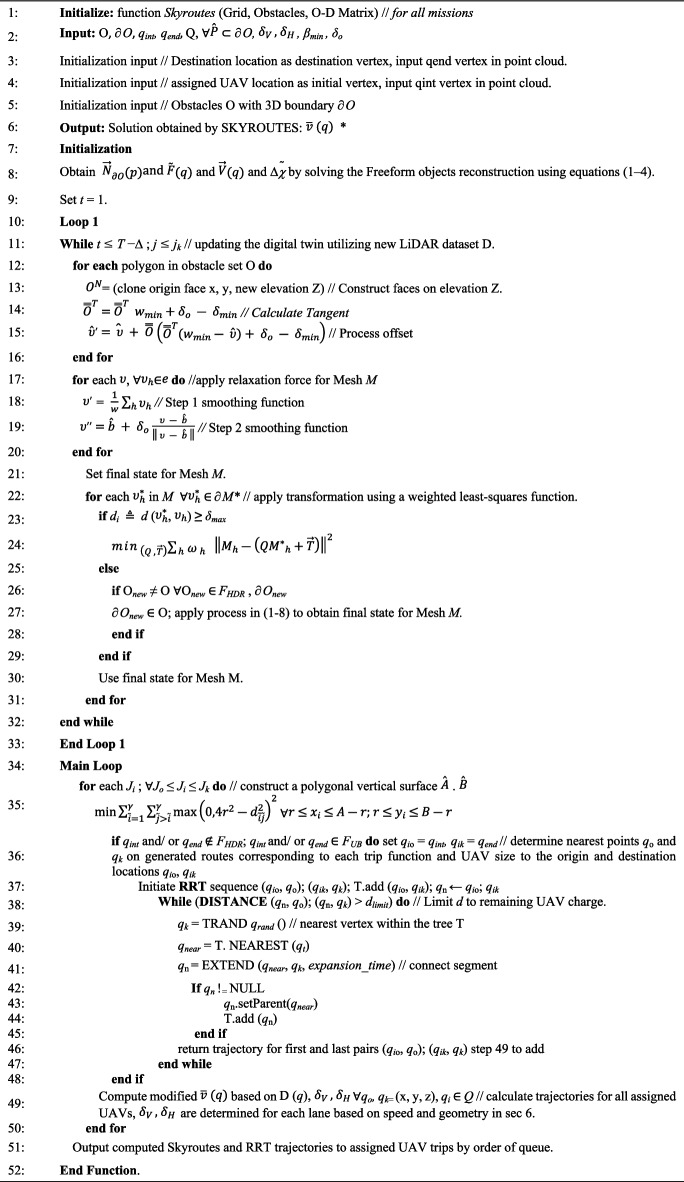
**Algorithm 1.** Pseudocode for the Robust Skyroutes Algorithm

## Trip generation, Cartesian routing, and UAV energy consumption

To test the operability and assess the efficiency of the proposed algorithm, a high-traffic load operation duration has to be simulated. An urban transportation simulation requires access to the specific location demand data. However, real-life georeferenced demand data is protected under different privacy laws. In this study, we model the origin and destination trips by adopting a realistic approximation from statistical prediction models that have been used in trip generation models and proved a high level of accuracy and robustness^22^.

To generate a heterogenous trip generation in terms of UAV size and trip nature (package delivery, flying taxi, or ambulance), we assume that the model follows a Poisson-distribution. The Poisson distribution is commonly used in various transportation demand modelling since it is considered an activity that will occur at a constant rate over a duration of time^87^. The mean variation is based on the simulation area census population density. The trip generation Eqs. (28–31) are outlined in the appendix for reference. The density map and the probability generation algorithm based on this Poisson distribution are coded in Python and overlaid on the city digital twin model. The resultant O-D matrix is the base for the TA. UAVs are assumed to start the trip by Vertical Takeoff (VTO) from the roof of the origin building mesh and ending the trip by Vertical Landing (VL) at the destination roof. To link the transportation tasks among generated O-D geolocations on the city digital twin model mesh generated in “Airspace discretization model description”, an area allocation and UAV assignment planning process is applied, however, TA does not fall within the scope of this study.

**Figure 12:**
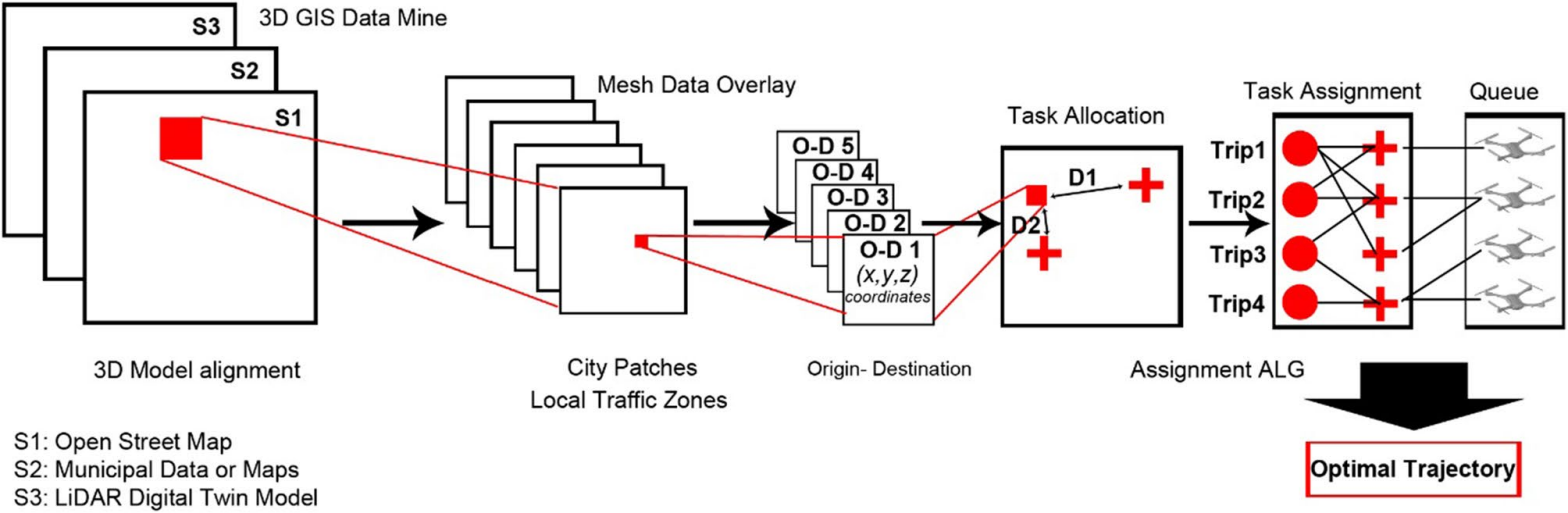
Proposed 3D GIS mining framework.

To assess the robustness of the proposed algorithm in this study, a single serving coverage area is considered, and each UAV is assigned one trip per timestep. Multiple randomized trip objectives, payload, duration, and travel distance determine the UAV size. The city digital twin is divided into clusters or volumetric patches according to several parameters including urban density and maximum building-footprint area. While for traffic and lane management, a first-come-first-serve queuing protocol is implemented. A full 3D GIS mining framework similar to neural networks is proposed and illustrated in Fig. 12. After the digital twin data is processed, the autonomous UAV trip generation and TA allocation loop is provided with pairs of coordinate points (latitude and longitude) via GPS link. The trips are generated based on the pre-explained Poisson distribution randomly to produce the full range of trips length and route complexity. The *Skyroutes* algorithm routes the trip and blocks the allocated lane segment $[\overline{v }(q\text{)]}$ at the utilized timestep *T* for other UAV trips.

**Figure 13:**
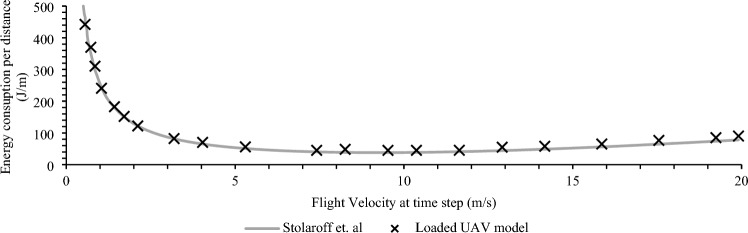
Experimental verification of calculation model.

The UAV lane-trajectories resulting from the algorithm depends on ${\delta }_{V }, {\delta }_{H }$ values. These values are determined for each lane based on lane designated speed, hence the resulting geometry as discussed by ElSayed and Mohamed^22,80^. To reach the optimal energy consumption and speed, the UAV motion is simulated based on the quadrotor physics. Mainly, the power divided on rotors that define the way the UAV moves and response, such forces, torque and thrusts are the key to UAV motion.

While UAV flight dynamics differ by airframe type, the main variants are fixed-wing and multi-rotor. Unlike fixed-wing UAVs, a multirotor possesses more than two rotors with hovering capabilities, such as quadrotors and hexarotors. For Quadcopters, a multirotor is controlled by altering the relative speed of each rotor to adjust the thrust and torque produced by each propeller opposing drag vector about the center of rotation. The four propellers are positioned at the corners of a square chassis as a pair of rotating blades. The motion equations are explained in the appendix (Eqs. 32–46).

The calculated energy consumption in Eqs. (35–40) aligns with real-world experimental results given the same input parameters for an experimentally verified model for a loaded quadcopter from the literature in Stolaroff et al.^88^ and ElSayed and Mohamed^22^. Results illustrated in Fig. 13 show high agreement at lower velocities, with a 5% discrepancy at higher velocities due to discrepancies in model assumptions.

At flight velocities over 3 m/s, translational lift increases the power efficiency significantly. While the speed profile will vary based on the path geometry and the status of the UAV (loaded or unloaded), to achieve the best energy efficiency velocities are maintained above 10 m/s and below 20 m/s in the generated lanes to maintain the viable route while capitalizing battery utilization.

Although the *Skyroutes* algorithm creates the main trajectories, the last trip leg in the local traffic zones *F*_*UB*_ operate under a full-mix airspace pattern. Due to the low traffic density, this airspace hardly needs regulation, the cartesian discretization is utilized to find the first/last leg of a trajectory using any of the literature solving algorithms. In this study, we utilize a modified Random Reduction Tree (RRT). A basic RRT works through three functional procedures, firstly the ‘generation’, finds by calculation a path between *q*_int_ ‘starting vertex’ and *q*_*end*_ ‘destination’ vertex which is obtained by growing a random search tree. The tree branches out in a highly dimensional environment to search for possible vertices from the starting vertex towards the destination with bias along the direct connector vector. Secondly, ‘the expansion’, a random vertex *q*_rand_ is picked and a line segment ‘edge’ is interpolated between the new vertex and last tree vertex in the list. With each iteration, a new edge and vertex are added to the path and the tree list expands till the destination vertex becomes a part of the tree. This leads to the third and final process ‘termination condition’.

Although highly successful, this basic calculation method becomes memory consuming. Moreover, the convergence rate is relatively slow in cases of complicated path planning where the chance of collision is significantly high in an obstacle rich environment such as our case study. Utilizing the A-star algorithm approach, in this case, amends this downfall and ensures the solving tree is only considering the most relevant areas of the point cloud tree. Whereas in a typical RRT the whole model space is populated with a point cloud and is considered for the solution. On the other hand, the Astar transforms the search into a function of the range of vertices confined along the direct path between *q*_int_ and *q*_sos_, this becomes the point populated domain, and the function is formulated as follows:

21 $${\dot{q}}_{t}\text{ = }f \, \left({q}_{t}-1,{\dot{u}}_{\text{t}}-1, {v}_{\text{t}}\right)$$

22 $${D}_{t}\text{ = }h \, \left({q}_{t}, {v}_{\text{i}}\right)$$

where ${\dot{q}}_{t}$∈ *Q* is the initiation point vector; ${\dot{u}}_{\text{t}}$∈ *U* is the destination vector; *v*_t_ is a random process disturbance appropriately determined; *D*_t_ is the measurement vector and *q*_i_ is a random component of the *q*_t_ tree.

Similar to Dijkstra algorithm, the Astar algorithm contains an open list of the potential waypoints *q*_free_ vertices, in addition to a closed list of all the visited vertices and a simple cost equation for solving as follows:

23 $${T}_{i}\text{ = }{C}_{i}+ {E}_{i}$$

where subscript *i* stands for the vertex call number in the RRT; *T*_i_ is the total cost (path length to minimize from *q*_int_ to *q*_*end*_) similar to Eq. (15); *C*_i_ is the current ith cost from *q*_int_ to current vertex; *E*_i_ is the estimated cost of *i*th vertex from the current vertex to the *q*_*end*_ destination vertex. To simplify the solution and solving time, the algorithm is also written and compiled in Python.

## Case study, results, and discussion

**Figure 14:**
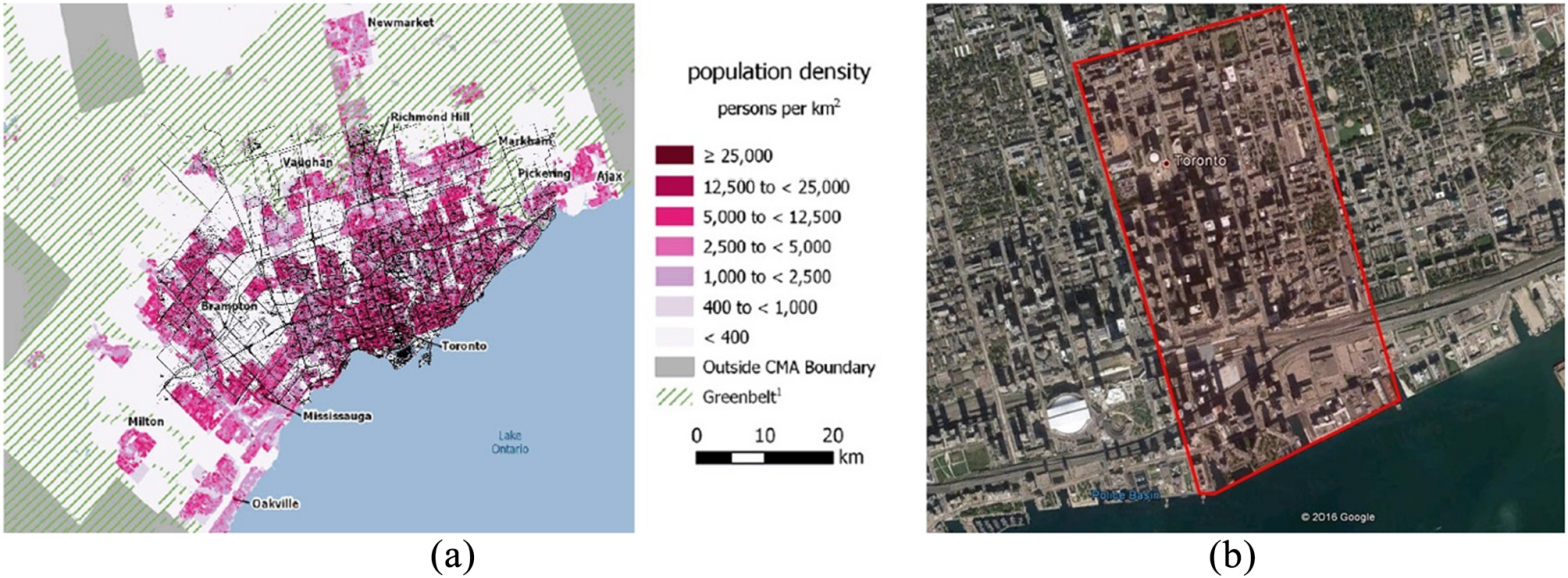
(**a**) Macro scale GTA census map^89^. (**b**) Aerial image of the study area (marked in red) and city context^90^. Source for (**a**): https://www150.statcan.gc.ca/n1/en/catalogue/16-508-X; Source for (**b**): Google maps with a Microsoft word 365 markup edit, google.com/maps.

A case study of a real 3D urban area in the densest section of the City of Toronto, one of the biggest urban centers in North America, was used to test the model and algorithm. With a population density of 4149.5 p/km^2^, occupied with various commercial, residential, and infrastructure buildings, the area represents a typical example of a mixed-use urban center. The area is covered in clusters 50 and 51, East York Patch, with an approximate area of 3.16 km^2^, presented in Fig. 13b. It features dense high-rise buildings and airfields, which can be complex for other discretization methodologies Fig. 14b.

Based on the Canadian census population density maps (Fig. 13a), a base-case scenario operation model was conducted as outlined in the methodology section on six downtown three-digit postal code areas and the associated geocoded information. Only local trips within the study area are modeled given the UAV range limitations on a single charge roundtrip. Results of the daily O–D Poisson generation are reported in Table 3, while peak-hour (5 p.m.) trips (around 1138 trips) are visualized in Fig. 14a. The ED is shown in green lines for UAV trips.

We carried out the *Skyroutes* discretization and cartesian discretization for performance comparison. In this section, we compare the results between the two schemes on three fronts, namely, Airspace utilization represented by the utilization factor (U) metric; hazard mitigation factor represented by the risk factor (ξ) metric; and Kinematic and energy efficiency represented by the total change in Euler angles (RAD). Considering the computational performance, results in Table 4 show the proposed Algorithm is comparatively similar to the solution time of Rapidly-exploring Random Trees (RRTs) and significantly better compared to Dijkstra. A thorough discussion on the performance has been explained for complex environments with dynamic elements in a previous study^81^. We have also illustrated the computational effectiveness by a simple test run against the Gurobi solver demonstrating significantly low solution time^91^.

**Table 3:** Comparison of computational performance.

Element		Settings	Algorithm				Unit
			Base model	Dijkstra	Modified RRT	Skyroutes	
			
1-Digital-Twin	Solution image						
2-Simulation parameters	Solving base	Type of path generation distance between vertices solution time		B-Spline 2 221	Bezier Curves 2 89	Lanes N/A 74	Sec
3-Processing power		Processor: Intel Core I9 CPU with single core utilization of 2.20 GHz; Memory: 64 GB

**Table 4:** Results of the O–D trip demand model.

Discretization method	Cartesian and proposed *Skyroutes*
Service area	3,663,251 m^2^
Poisson λ parameter	Six 3-digit allocations
Average trip distance (min, max)	811.26 (24.32, 2059.35) m
Average ED between destinations (spread)	52.1 m
Mission count (trips)	1138
Longest route ED	2059.35 m
AM peak	9–10 a.m.
PM peak	4–6:30 p.m.

### Geofencing results compared to cartesian discretization

**Figure 15:**
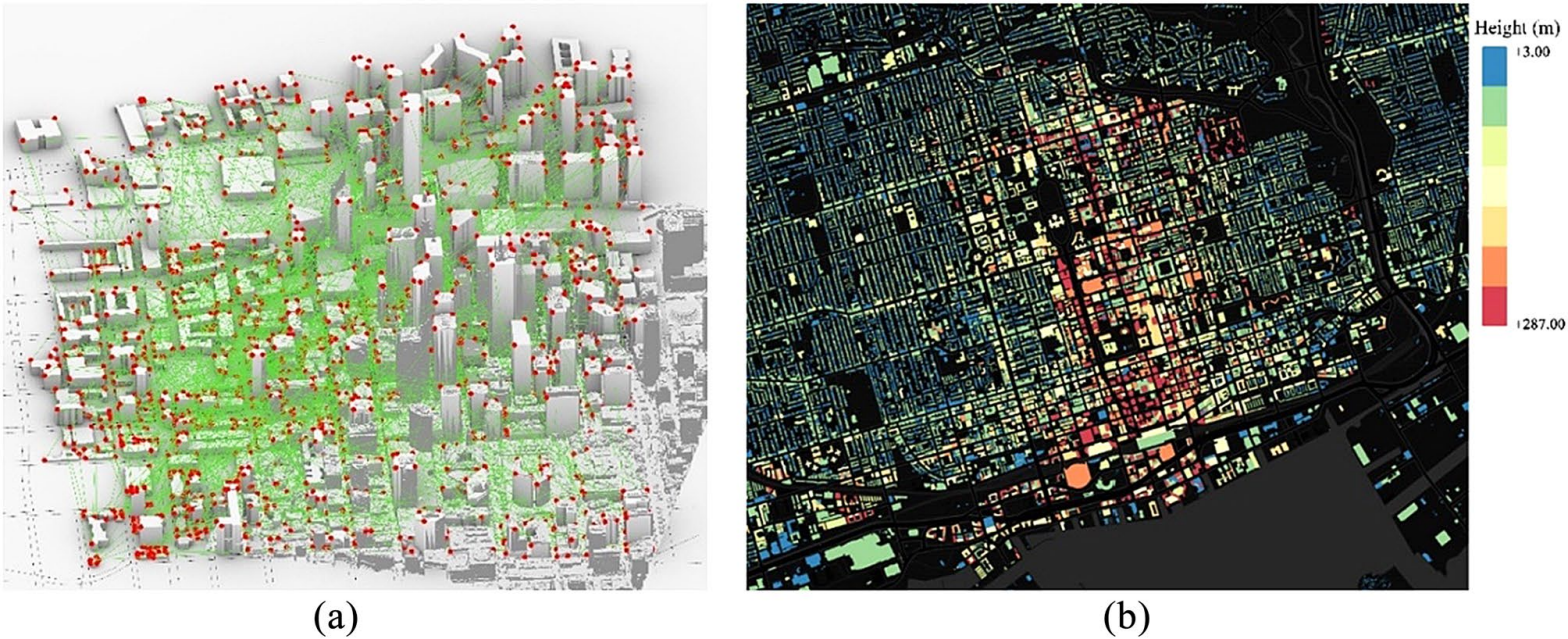
(**a**) O–D points (in red) ED of peak-hour trips (in green). (**b**) Study area in old Toronto showing height distribution of structures in airspace. Source for (**a**): Authors created by Rhinoceros 3D, version 7, rhino3d.com; source for (**b**): Authors created by Rhinoceros 3D, version 7, rhino3d.com.

In the case study, airspace between 30 and 150 m (100 m for strict regulations) is considered for the UAV traffic. Starting with city obstacle mesh M, the airspace was first divided into the two volumetric sets *F*_*HDR*_ and *F*_*UB*_ described in the methodology section. Then, the Skyroutes morphology at ${\delta }_{H }=1$ yielded 40 levels of keep-in lanes flowing along the *F*_*HDR*_ shown in Fig. 15a. Similarly, the airspace was discretized into a three-dimensional regular grid of 3 m and 5 m for strict regulations, resulting in a 440 × 360 × 40 α-ball Cartesian grid in a dual geofence Fig. 15b.

**Figure 16:**
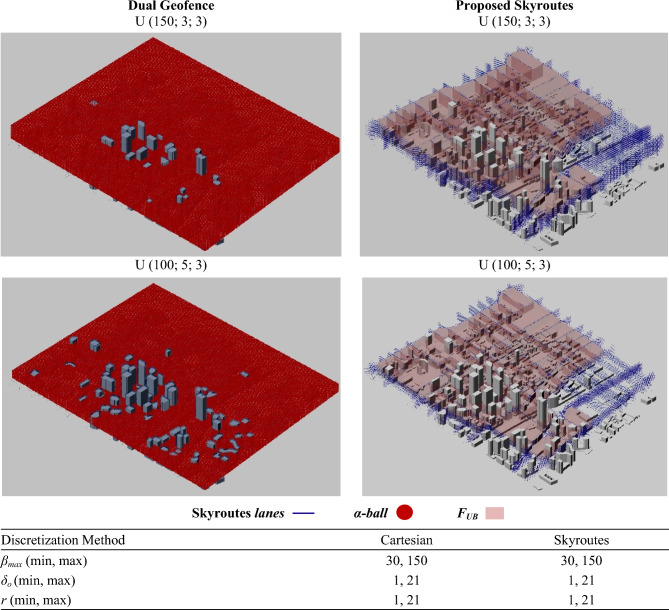
(**a**) Cartesian grid discretization, keep-in (red), Keep-out (blue). (**b**) Skyroutes discretized airspace, lanes (blue), F_UB_ (red).

To compare the results across both methods, we use a utilization factor U (*β*_*max*_*; δ*_*o*_; r) for cartesian discretization, where (*δ*_*o*_) is the minimum clearance distance from the nearest obstacle; and r is the keep-in radius for UAVs; (*β*_*max*_) is the maximum flight altitude dictated by the applicable flight policy. For the Skyroutes algorithm, O-Ds without major road access utilize the *F*_*UB*_ for the first/last leg in the trip connecting to the lanes. Figure 15a highlights the results of maximized utilization of airspace with lean flight policies, the α-ball utilization coverage in 3D is U (100; 5; 3) = 88.1% and U (150; 3; 3) = 93.1%, respectively. This is due to the added airspace volumes in the cartesian discretization, the same utilization gains are reflected in the Skyroutes algorithm results as the maximum flight altitude *β*_*max*_ increases, hence adding more lanes for UAVs.

These results highlight several observations, first, the benefits of using the Mesh M generated from LiDAR point cloud (lines 1 to 33 in Algorithm1) as a more precise tool in airspace capacity estimation as compared to other 2D methods in the literature where capacity is calculated by a series of horizontal 2D slicing planes. Second, the benefits of dual (keep-in and keep-out) geofencing technique allow higher control to apply airspace flight policies and NFZs.

While the airspace utilization increases significantly in both discretization methods with leaner flight policies (5% for cartesian and 10% for Skyroutes), however, the cartesian discretization shows higher sensitivity to the *δ*_*o*_ value, as compared to the Skyroutes method, which relies heavily on the *β*_*max*_. The proposed Skyroutes algorithm shows a higher level of robustness in eliminating the inconsistency of airspace utilization variance with altitude through a linear behaviour compared to an exponential behaviour in the cartesian morphology. By eliminating bottlenecks as UAVs propagate in lower airspace for main trajectories in the *F*_*HDR*_, the exponentially higher estimates of utilization in *F*_*UB*_ indicates higher UAV traffic above buildings.

This proves the substantial benefits of using the Skyroutes algorithm over the cartesian method in airspace capacity estimation that further aligns with the AAM civil airspace safety and privacy objectives.

Similarly, Fig. 16 shows the results of airspace capacity utilization and loss in terms of different *β*_*max*_ at variable values for *δ*_*o*_ and r. The results’ matrix heatmaps show the robustness of the proposed methodology in estimating airspace capacity with extreme precision. Overall, the effect of geofencing was most restrictive in the lower altitude levels, when *β*_*max*_ ≤ 60 due to the high-density obstacles. However, the impact on cartesian discretization is significantly more severe compared to the impact on the Skyroutes discretization. Also, generally it can be noticed in all altitudes and across different *δ*_*o*_ and r combinations, the Skyroutes discretization yields a higher airspace capacity. This is due to the advantage of using the cylinder/circle packing subroutine Eqs. (14–15) to fit more lanes as compared to the cartesian division.

**Figure 17:**
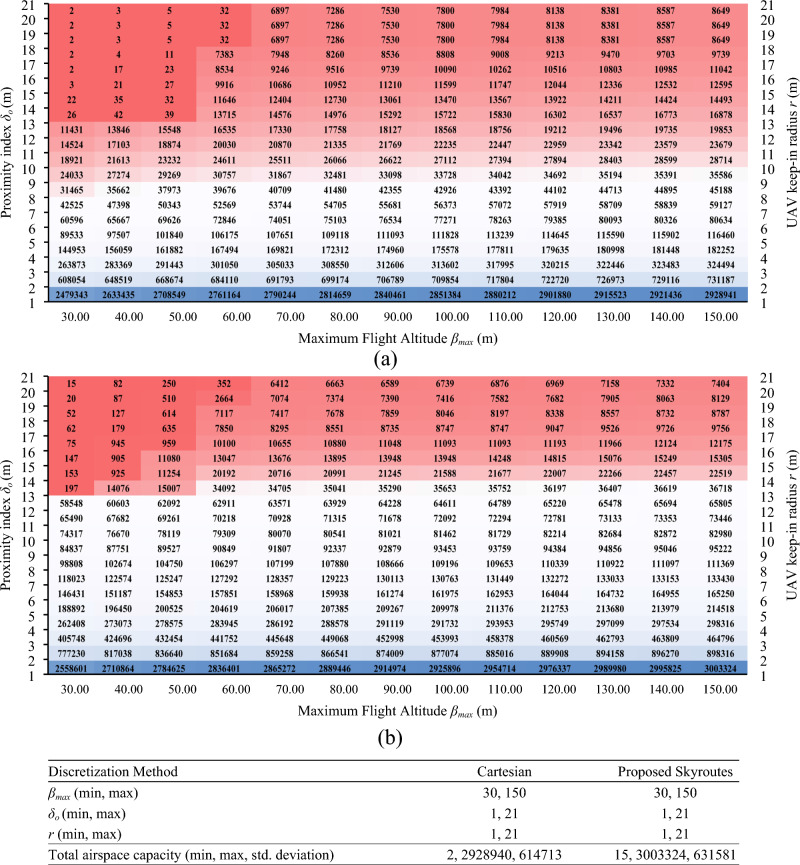
Discretization airspace capacity matrices. (**a**) Cartesian discretization airspace capacity matrix. (**b**) Proposed Skyroutes discretization airspace capacity matrix.

Furthermore, due to the island effect in cartesian discretization if an airspace patch has less than 25 m (this is dominant in lower altitudes with higher *δ*_*o*_ and r combinations) of travel range, the entire discretized patch is not considered in the capacity estimation. This is not the case for Skyroutes since the lane discretization is performed in 3D, which sometimes allow only a narrow path in higher altitudes to utilize this entrapped discretized airspace void in lower altitudes. The matrices presented in Fig. 16 can guide policymakers in finding the regulation combinations to achieve a desired level of civil airspace utilization, and to evaluate the operational feasibility based on trade-offs between *β*_*max,*_* δ*_*o*_, and r.

Airspace utilization and loss matrices prove more efficient and robust in airspace capacity estimation as compared to 2D graphs and curves. Matrices highlight the severe impact of higher *δ*_*o*_ and r combinations ≥ 10 m in lower airspace levels *β*_*max*_ ≤ 60. This highlights the sensitivity to altitude in tighter urban scenarios such urban centers and high-rise downtown areas. It also highlights the flexibility of dual geofencing (keep-in and keep-out) in determining the safe airspace utilization. Whereas higher airspace altitude *β*_*max*_ ≤ 60 shows a slightly greater advantage to cartesian discretization over Skyroutes, results show a 10% increase in airspace capacity estimation as the free-mix airspace model is applicable. In general, digital-twin volumetric 3D approach shows robust capability to assess airspace capacity with different policy permutations.

### Air traffic safety and hazard mitigation performance

In this section, we present the differences in airspace safety and hazard mitigation between cartesian and Skyroutes discretization. While, noise reduction is illustrated by visualizing the UAV trajectories around the study area, safety is defined by a risk factor (ξ), which is the proximity of the UAV trajectories to moving obstacles and other UAV trajectories or the possibility of the UAV derailing from the designated lane or trajectory. Figure 17 shows Cartesian and Skyroutes discretization airspace UAV trajectories at 5 pm for the study area. To assess the robustness of the proposed algorithm, we utilize the modified RRT* as well as several relevant UAV 3D routing and trajectory optimization literature from Table 2 for each UAV trip and only use the most efficient results for the cartesian method.

**Figure 18:**
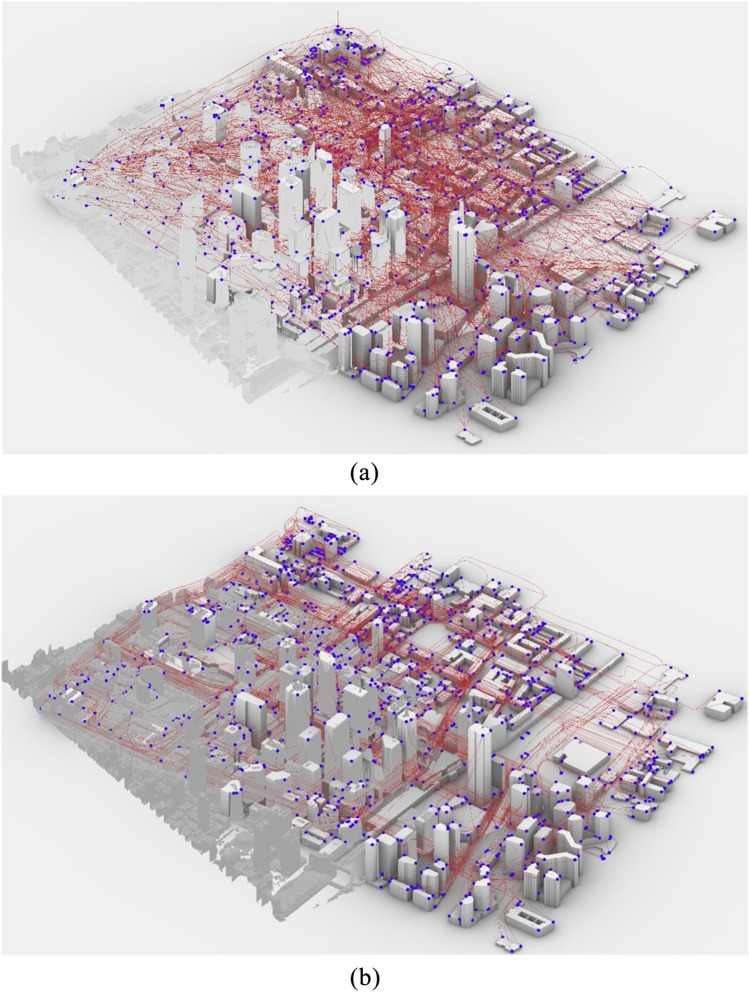
Discretization airspace trip trajectories. (**a**) Cartesian discretization airspace trip trajectories 5 pm. (**b**) Proposed Skyroutes discretization airspace trip trajectories 5 pm.

The results show several trends, first, the significant difference in noise reduction, as UAV trajectories avoid the utilization of airspace above urban blocks in *Skyroutes* trajectory optimization versus the cartesian trajectories. This is with the exception of take-off and landing (last leg) performed as part of the TO task and amalgamated to the total given mission trajectory to avoid the outlined accident risk. Second, the *Skyroutes* results show a significant airspace order as compared to cartesian methods due to the aggressive use of the airspace above urban blocks F_UHD_ to achieve the shortest trajectory possible. The proposed algorithm regulates all trajectories in the F_HD_ volume mostly aligning with the study area’s major road network starting from the minimum flight altitude (*β*_*min*_) up to the maximum flight altitude (*β*_*max*_).

**Figure 19:**
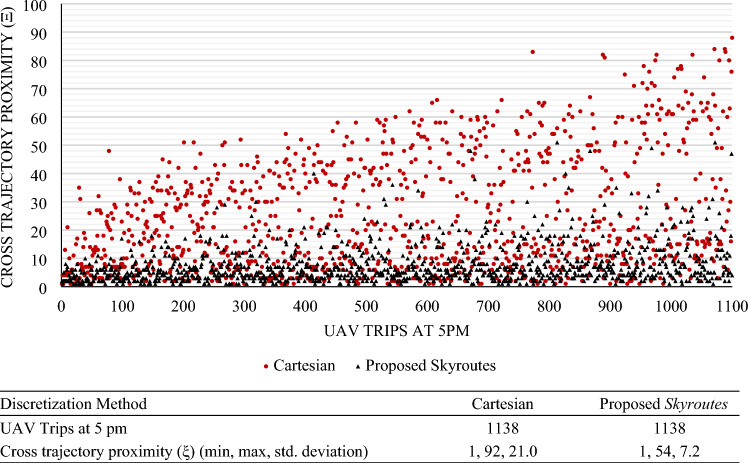
Cross trajectory proximity results.

Further, Fig. 18 shows the results of cross trajectory proximity for both discretization methods. *Skyroutes* algorithm shows a significant reduction in the instances of cross trajectory proximity where trajectories are in closer proximity (distance between trajectories at any point is < 3 m or intersecting) at a critical time window ≤ 30 s. The lane geometrical design and timestep queuing method allows optimizing the trajectories by spacing them whenever possible mitigating multiple trajectory collision. Along the same lines, Fig. 19 shows the significant reduction in the trajectory Euler transformations (explained in Fig. 7) which ensures the integrity of the payload within the keep-in geofence and reducing the risk factor (ξ) of UAV derailing from the designated trajectory.

**Figure 20:**
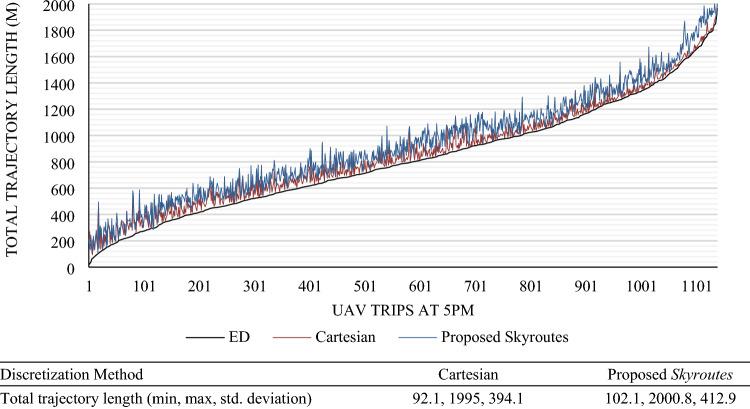
Total trajectory length results.

### Kinematic and energy efficiency

The results show up to 30% lengthier trajectories (in 8.2% of the cases), and up to 10% increase for the rest of the trajectories for the *Skyroutes* discretization as compared to cartesian discretization, Fig. 19. This increase in route length comes from the instances where origins or destinations are deep in the congested areas of the airspace or from the need for multiple lane changes due to the queue. However, the overall energy consumption is up to 50% lower in more than 60% of the trips for the proposed *Skyroutes* discretization and trajectory optimization algorithm.

This is due to the consistency of the trajectory as stretches of straight lines in the keep-in lanes allow UAVs to maintain the maximum efficient speed of 20 m/s without the need for deceleration on maneuvers as in the case with the cartesian trajectories. This is illustrated in Fig. 20 as the total change in Euler angles along the UAV trajectories. Less change in trajectory angular motion means significant reduction in rotor torque changes allowing the UAVs to travel a longer distance at the optimal discharge rate and decreases the depletion of charge^80^.

**Figure 21:**
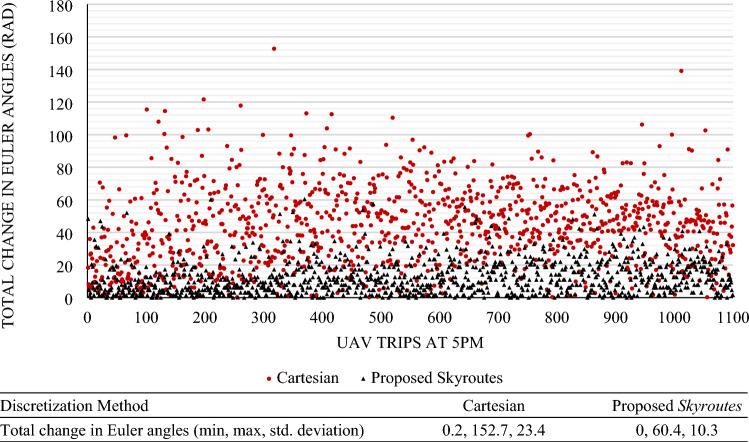
Total change in Euler angles along UAV trajectories results.

## Conclusions and future studies

In this study, we proposed a novel autonomous Advanced Aerial Mobility (AAM) system for high density city centers that dynamically discretizes the viable airspace into UAV trajectories. By incorporating the city’s digital-twin model through interpolating LiDAR data and a dual keep-in and keep-out geofence, our method expands the functionality beyond airspace capacity assessment to test different flight policies and measure the tradeoffs between them. Furthermore, the proposed algorithm converges energy-efficient UAV trajectories while minimizing the safety hazards and sound pollution.

Since UAVs are assumed to be automatically piloted by an embedded mission control system, in a heterogenous fleet situation or a multi-user traffic control narrative, the onboard flight controller on each UAV requires a pre-planned trajectory with multiple contingencies (alternative routing) for specific mission assignment and teamwork logistics. This highlights the benefits of the proposed *Skyroutes* with multiple lanes rather than a full-mix airspace morphology.

In the hypothetical case of a complex urban scenario, we demonstrated that the digital-twin model is crucial for the precision and safety of pre-planned UAV trajectories. The proposed *Skyroutes* algorithm was able to identify narrow urban corridors and maximize the airspace capacity up to 10% increase in a severely restricted airspace by connecting isolated airspace volumes through a circle packing sub-routine as compared to cartesian discretization, which was unable to tackle this challenge efficiently. A case study of Toronto city center, Canada illustrated the robust capabilities of the proposed algorithm in a real 3D environment.

The cartesian airspace discretization allows the applicability of a variety of trajectory optimization algorithms in a full-mix airspace morphology, while the *Skyroutes* capitalizes on the energy-efficient trajectories and regulating the airspace traffic management through combining several airspace morphologies. For cartesian discretization, on the one hand, a tight mesh (waypoint vertices) results in a slower and more complicated graph-solving task due to the significantly large size of the solving domain. On the other hand, a wider mesh results in less available solutions and more unutilized tight spaces within the dense city urban form where spacing between the towers can be less than three meters wide. The application of dynamic meshing method in digital-twin models shows the agility of capturing urban details, where building protrusions, setbacks, construction tools (such as cranes) and other architectural features such as street vegetation and landscape elements within the urban setting are taken into consideration. This allows the solving algorithm to diminish collision chances and relieve the reliance of on-board sensors. Also utilize tight spacing within the study area while avoiding the probability of algorithm’s solution errors that could cause obstacle collisions. The *Skyroutes* discretization is more adaptive and can deliver significantly higher airspace usability coupled with more challenging capabilities especially in highly restrictive airspace.

The proposed *Skyroutes* algorithm successfully demonstrated the ability to analyze the flight policy combinations in the case study. The precision in estimating the airspace capacity showed high sensitivity to the variables, which suggests that the current approach that relies on 2D or cartesian discretization measures needs further evaluation for effective urban UAV operations. The proposed algorithm illustrated the difference in safety and energy-efficiency of the converged trip trajectories. The results also show significant improvements over cartesian discretization, the overall energy exerted by UAVs to overcome a lengthier trajectory is outweighed by lower torque changes, lower energy consumption, and lower noise levels avoiding urban airspace over inhabited areas. Furthermore, reduced cross-trajectory proximity and the proposed lane change sub-routine allows higher coordination and safety by providing alternate routing in case of disruptive events.

One of the possible limitations of the proposed algorithm is the universal applicability on any urban scenario. Since the urban density and city morphology adds limitations for every unique situation. If a civil authority seeks a specific flight policy that can apply to all cases of diverse geospatial complexity to operate autonomous civil UAV flights, it can either be prone to higher risk factors or severely restrict the viable airspace and UAV size/type choice. While the proposed method can efficiently determine the adequate policy combination (βmax; δo; r), simulations are inevitable for precise results. In addition to evaluating the airspace usability, our approach generates a crucial dataset to model civil airspace in 3D. Identifying the continuity of trajectories will be necessary for structured urban airspace design and path planning. This will strategically serve developers, planners and decision-aiding authorities such as the Model Aeronautics Association of Canada (MAAC) to operationalize UAVs in the near future. The integration of smart, sustainable and autonomous robotics for transportation in smart cities represents a silver bullet solution for the aforementioned challenges. In the future, we plan to add more uncertainties such as wind dynamics to add robustness to the proposed airspace discretization algorithm and increase energy-efficiency.

### Supplementary Information

Supplementary Information.

## Data Availability

The LiDAR and the 3D city model utilized in this study are available through the Toronto City Data Catalog at https://mdl.library.utoronto.ca/collections/geospatial-data/toronto-lidar-2015. https://www.toronto.ca/city-government/data-research-maps/open-data/.

## List of symbols

$O$: Set of solid obstacles in LiDAR data
$\partial O$: Solid boundaries of obstacle
*χ*_*O*_: Indicator function of $O$
${\overrightarrow{N}}_{\partial O}\left(u\right)$: Inward surface vector normal at point *u*
$\widetilde{F}\left({f}_{0}\right)$: Gaussian smoothing filter
*S*: Oriented batch matrix points
$\widehat{P}$: Surface Patch at point *s*
$$\vec{V}$$: Vector field
$\nabla$: Vector Divergance operator
*ϐ*: Octree notation
*F*_*o*_: Associated node function at *o*
$\vec{V}(q)$: Gradient field indicator function
*Ω(s)*: Octa closest depth nodes
$\alpha_{o,s}$: Trilinear interpolation weights
*δ*_*o*_: Flight policy buffer distance (minimum clearance)
$\widehat{\upsilon }$: Polygon center projection
${\upsilon }{\prime}$: Polygon surface sample
*w*_*min*_: Minimum point on polygon
$\overline{\overline{O}}$: Minimum normal vector to the tangent polygon plane
$\widehat{b}$: Mesh base point
*Ꞙ*: UAV viable airspace volume
*Q*: Rotation matrix
*ω*_*i*_: Weighing variable
$$\vec{T}$$: 3 × 1 Translation vector
*N*: Matrix of B-spline basis functions
$\overline{P }$: Matrix of curvature degrees
g: Acceleration due to gravity
*β*_*min*_: Minimum flight altitude
*β*_*max*_: Maximum flight altitude
*F*_*UB*_: Urban block airspace
*F*_*HDR*_: High density routes airspace
$$\overset{\lower0.5em\hbox{$\smash{\scriptscriptstyle\smile}$}}{S}$$: Polygon segment
${q}_{obs}$: Obstacle geometrical center
D(*q*): Lane disruption function
*B*_obs_: Effective matrix of obstacles
$\widetilde{v}$: Initial lane vector flight velocity
$\overline{\overline{v}}$: Traversing UAV velocity
*M*: Model mesh
*δ*_*V*_: Lane proximity for vertical policy parameter
$u$: Solid boundary points *u* ∈ *∂O*
*s*: Matrix points *s* ∈ *S*
*f*: Field points
*o*: Octree nodes *o* ∈ *ϐ*
*e*: Mesh polygons counter
*h*: Polygon vertices counter *h* ∈ *w*
$\widehat{k}$: Total number of trajectory vertices
$\widehat{i}$: Trajectory vertices counter
*q*: Trajectory vertices
*γ*: Total number of lanes
*k*: Destination section counter
v: Angular velocity
$\dot{\theta }$: Angle time derivative
$$\tau$$: Torque
*C*_*τ*_: Constant of torque
*I*: Electric current input
*V*: Rotor voltage feed
*R*: Coil resistance
*C*_p_: Proportionality constant
P: Power consumption
$$T_{h}$$: Rotor thrust
*v*_L_: Loft velocity
*ρ*_air_: Density of air
*a*: Area covered by each rotor
C: Overall constant
$\dot{v}$: Angular velocity vector
$$I_{n}$$: Inertia
*F*_d_: Drag force
$$m$$: Mass
$\sigma_{f}$: Horizontal flight angle in roll axis
$\rho_{{{\text{ref}}}}$: Reference lane curvature
$d_{l}$: Target lane change longitudinal distance
${\dot{\text{x}}}_{\max }$: Maximum UAV lateral acceleration
*δ*_*H*_: Lane proximity for horizontal policy
p (q): Perpendicular vector to the UAV path
t (q): Tangential vector to the UAV trajectory
MA: Matrix of rotation within both body and inertial frames
*ф*,* θ*,* ψ*: Pitch, Roll, and Yaw angles
