# Supplementary material for: Robust digital-twin airspace discretization and trajectory optimization for autonomous unmanned aerial vehicles
Source: Sci Rep. 2024 May 31;14:12506. doi: 10.1038/s41598-024-62421-4 (PMC11143258; doi:10.1038/s41598-024-62421-4)
Supplement: Supplementary file 1 — Supplementary Information. [file 41598_2024_62421_MOESM1_ESM.docx]

# Appendix

## Appendix-I

Given the set of solid obstacles $O$ with $3D$ solid boundary ∂ *O*, the gradient of the smoothed indicator function is calculated through smoothing the surface normal field at points *f* by:

∇(χ*_O_ .* $\tilde{F}$ )*(*$f_{0}$*)* ***=*** $\int_{\partial O} \tilde{F}_{u}\left( f_{0} \right)\vec{N}_{\partial O}\left( u \right)du$ (1)

Where χ*_O_* is the indicator function of *O*; $\vec{N}_{\partial O}\left( u \right)$ is the inward surface vector normal at point $u$ *∈* ∂ *O*; $\tilde{F}\left( f_{0} \right)$ is a Gaussian smoothing filter translating $point f_{0} to point u$. Since surface geometry is unknown, we can approximate the surface integral over surface patches $\hat{P}$ using discrete summation from the set of oriented batch matrix points $S (s\in S)$ to divide ∂ *O*, the value at point sample $s.u$ is scaled by the area of $\hat{P}$:

∇(χ*_O_.* $\tilde{F}$ )*(* $f$*)* ***≈***$\sum_{s\in S} \left| \hat{P} \right|\tilde{F}_{s.u}\left( f \right)s.\vec{N}$≡ $\vec{V}\left( f \right)$ (2)

Now that we have the vector field $\vec{V}$For the point cloud data, we used an adaptive “crawling” octree (a data tree with three branches) from each sample point to represent the implicit function and solve for the least-squares approximate solution. First, the divergence operator is used:

Δ$\tilde{}$ = ∇.$\vec{V}$ (3)

Then, we use the positions of the sample points to define an octree *ϐ* with an associated unit-integral node function $F_{o}$ for each node $o\inϐ$. Applying this to the product of (2), we can get an approximation to the gradient field indicator function by:

$\vec{V}\left( f \right)$ ≡$\sum_{s\in S} \sum_{o\in\Omega\left( s \right)} \alpha_{o,s}F_{o}\left( f \right)s.\vec{N}$*; for each* $\Omega\left( s \right)$ *Euclidean closest node in tree depth to s.u* (4)

Where $\alpha_{o,s}$, are the trilinear interpolation weights for the nodes; $\Omega\left( s \right)$ are the eight set-depth nodes closest to point sample *s.u*. Assuming constant batch areas and having an acceptable approximation for the defined vector field $\vec{V}$, we can extract the iso-surfaces from the indicator function, we utilize an adaptation of the Marching Cubes method to octree representations ^[85,86]^. The method source code is available in C language by Paul Bourke ^[87]^.

## Appendix-II

The generation algorithm calculates the probability of $X$ occurrences by:

*P(X)* ***=*** $\frac{\lambda^{n}e^{-\lambda}}{x!}$ (28)

Assuming the transportation demand over time interval $t$ (one day) and the expected number of daily trips $n, e$ is Euler’s number, equal to 2.71828. We can rewrite equation (28) and extrapolate the probability of *n* occurrences in the interval *t* by:

*P(n;t)* ***=***$e^{-\lambda t}$ $\frac{{(\lambda t)}^{n}}{n!}$ (29)

*μ = λt* (30)

Where is the expected number of trips taken from the estimated 0.08 parcel per person per day above, the final equation is expressed as:

*P(n)* ***=***$e^{-\mu}$ $\frac{\mu^{n}}{n!}$*; for n = 0, 1, 2….* (31)

## Appendix-III

For the polygonal surface of radius $\breve{r}$ with $\breve{n}$ sides, each segment $\check{S}$ can be described by:

$\check{S}=\left\{ \left( \breve{x},\breve{y} \right)\in\mathbb{R}^{2},a\breve{x}+b\breve{y}=c,\breve{x}_{0}\leq\breve{x}\leq\breve{x}_{1},\breve{y}_{0}\leq\breve{y}\leq\breve{y}_{1} \right\}$ (11)

$f_{n}\left( \breve{x}+\breve{i}\breve{y} \right)=|\breve{r}\check{S}_{\breve{n}}-\left| \breve{y} \right|\left| -\left( \breve{r}sin(\pi/\breve{n})-\left| \breve{y} \right| \right) \right|+\left| \breve{x}-\breve{r}cos(\pi/\breve{n}) \right|$ (12)

$\prod_{\breve{k}=0}^{\breve{n}-1} f_{n}\left( e^{-\frac{2\breve{i}\breve{k}\pi}{\breve{n}}}\left( \breve{x}+\breve{i}\breve{y} \right) \right)=0$ (13)

## Appendix-IV

The quadrotor UAV moves based on the body frame or rotor axes vectors in the $x, y$ and $z$ directions, which deviates from the inertial frame defined by gravity in the negative z-direction as shown in Fig. 7 in the manuscript. All rotors are assumed to be brushless identical electric motors, ($\dot{\theta}$) is the time derivative for the pitch, roll, and yaw angles of the body frame $\left( ф, \theta, \psi\right)^{T}$. The angular velocity ($v$) is defined as a rotational axial vector, and ($\mathrm{MA}$) is the matrix of rotation within both body and inertial frames of the UAV ^[88]^, accordingly both can be obtained by ^[76]^:

$v=\left[ \begin{matrix} 1 & 0 & \text{-}\text{ }s_{\theta} \\ 0 & c_{\varphi} & c_{\theta}s_{\varphi} \\ 0 & \text{-}\text{ }s_{\varphi} & c_{\theta}c_{\varphi} \end{matrix} \right]\dot{\theta}\text{ , }\dot{\theta}\text{ ≠ }v$ (32)

$\text{M}\text{A}\text{ = }\left[ \begin{matrix} c_{\varphi}c_{\psi}\text{- }c_{\theta}{s_{\varphi}s}_{\psi} & \text{- }c_{\psi}s_{\varphi}\text{- }c_{\varphi}c_{\theta}s_{\psi} & s_{\theta}s_{\psi} \\ c_{\theta}c_{\psi}s_{\varphi}+c_{\varphi}s_{\psi} & c_{\varphi}c_{\theta}c_{\psi} \text{-}s_{\varphi}s_{\psi} & {\text{- }c}_{\psi}s_{\theta} \\ s_{\varphi}s_{\theta} & c_{\varphi}s_{\theta} & c_{\theta} \end{matrix} \right]$ (33)

The torque production and voltage are given by:

$\tau\text{=}\text{C}_{\tau}\text{ }\text{( }\text{I }\text{-}\text{ I}_{\text{idle }}\text{)}$ (34)

$\text{V}\text{ =}\text{I}\text{ }\text{.}\text{ }\text{R}\text{ +}{\text{ }\text{C}}_{\text{p}}v$ (35)

Where $\tau$ is the torque (N. m); $\text{C}_{\tau}$ is a constant of torque; $I$ is the electric current input (ampere); and $\text{ I}_{\text{idle }}$ is the current at an idle rotor. $V$ is the rotor voltage feed (volts); $R$ is the coil resistance (ohm); $v$ is the localized angular rotor velocity ‘rotational speed’ (RPM); and ${\text{ }\text{C}}_{\text{p}}$ is the proportionality constant of back electromotive force. We can obtain the power for low-resistance motors via:

$\text{P =}\frac{\text{ C}_{\text{p}}}{\text{C}_{\tau}}\text{ }\text{v}\text{ }\text{.}\text{ }\tau=F\frac{dx}{dt}$ (36)

Where P is the rotor power consumption to maintain the UAV flight (Watt). Since the system is assumed in this study to operate only under steady wind conditions, it is deductible:

$\text{P }\text{=}T_{h}\text{ }\text{.}\text{ }v_{L}$ (37)

Where $T_{h}$ is the rotor thrust (Newton); $v_{L}$ is the loft velocity at idle air position. Knowing that the thrust of the rotors is proportional to the square of angular velocity, it can be deducted through:

$T_{h} \text{= }{{\text{ }\text{C}}_{\text{v}}}^{2}\left( \frac{{\text{ }\text{C}}_{\text{p}}\sqrt{2 {. a . \rho}_{\mathrm{air}}}}{\text{C}_{\tau}} \mathbf{.} v \right)^{2}=\text{C}\left[ \begin{matrix} 0 \\ 0 \\ \sum{v_{i}}^{2} \end{matrix} \right]$ (38)

Where *ρ*_air_ is the density of air and equals an assumed average of 1.225 kg/m^3^ in this case; $a$ is the area covered by each rotor (m^2^). The overall constant is appropriately valued and denoted by $C$ for ease of calculation and coding. To apply the motion equations, all-controlling forces must be included in the matrix; hence, by deriving the rotational motion equations based on Euler’s equation:

$\tau\text{=}\text{ }\text{ }\text{(}I_{n}\text{ }\dot{v}\text{ }\text{+ }\text{v}\text{)}\text{ }\text{.}\text{ (}I_{n}\text{ }\text{v}\text{)}$ (39)

Where $\dot{v}$ is the angular velocity vector; $I_{n}$ is the inertia. From the rotor matrix $M$ given previously in equation (33), the linear motion can be deducted:

$motion ẋ_{i}=\left[ \begin{matrix} 0 \\ 0 \\ -mg \end{matrix} \right]+\mathrm{MA} \mathbf{.} T_{h}+{\text{ }\text{F}}_{\text{d}}$ (40)

Where $ẋ_{i}$ is the path of the UAV, g is the acceleration due to gravity and equals 9.81 (m/s^2^); m is the mass and $F_{d}$ is the drag force. From (39) and (40), assuming the quadcopter is symmetric about both the $x$ and $y$ axis, the equation can be reduced into a simplified inertial matrix as:

$\dot{v}=\left[ \begin{matrix} \dot{v}_{x} \\ \dot{v}_{y} \\ \dot{v}_{z} \end{matrix} \right]= \frac{\left( \tau\text{-}v\mathbf{.}\left( I_{v} \right) \right)}{I_{n}}$ (41)

$I_{n}\text{ = }\left[ \begin{matrix} I_{xx} & 0 & \text{0} \\ 0 & I_{yy} & 0 \\ 0 & \text{0} & I_{zz} \end{matrix} \right]$ (42)

By solving equations (41) and (42), the final formula can be expressed as:

$\dot{v}\text{ = }\left[ \begin{matrix} \tau_{\varphi} & {I_{xx}}^{\text{-}1} \\ \tau_{\theta} & {I_{yy}}^{\text{-}1} \\ \tau_{\psi} & {I_{zz}}^{\text{-}1} \end{matrix} \right] - \left[ \begin{matrix} \frac{I_{yy} \text{- }I_{zz}}{I_{xx}} & v_{y}v_{z} \\ \frac{I_{zz} \text{- }I_{xx}}{I_{yy}} & v_{x}v_{z} \\ \frac{I_{xx} \text{- }I_{yy}}{I_{zz}} & v_{x}v_{y} \end{matrix} \right]$ (43)

The formula given in (43) is coded in Python to mimic the motion of a UAV and provide the exact power to be dispatched to each propeller along the trajectory. The motion dynamics are also applied when a lane change is required. Firstly, the virtual reference lane from the Skyroutes algorithm is set as input, and then a lane change maneuver is applied, similar to EVs. In adaption of the lateral dynamics ^[89]^, the motion can be described by:

$\frac{d}{dt}\left[ \begin{aligned} &y_{f} \\ &\dot{y}_{f} \\ &y_{r} \\ &\dot{y}_{r} \end{aligned} \right]=\left[ \begin{matrix} 0 & 1 & 0 & 0 \\ a_{21} & a_{22} & -a_{21} & a_{24} \\ 0 & 0 & 0 & 1 \\ a_{41} & a_{42} & -a_{41} & a_{44} \end{matrix} \right]\left[ \begin{aligned} y_{f} \\ \dot{y}_{f} \\ y_{r} \\ \dot{y}_{r} \end{aligned} \right]+\left[ \begin{matrix} 0 & 0 \\ b_{21} & b_{22} \\ 0 & b_{32} \\ b_{41} & b_{22} \end{matrix} \right]\left[ \begin{aligned} \sigma_{f} \\ \rho_{\text{ref }} \end{aligned} \right]$ (44)

Where $\sigma_{f}$ is horizontal flight angle in the roll axis, $\rho_{\text{ref }}$ is reference lane curvature from $\delta_{V}, \delta_{H}, a_{ij}$, and $b_{ij}$are the pitch and roll UAV parameters, $y_{f}$and $y_{r}$are lateral displacement of the UAV from the onboard gyro to the reference lane, respectively. To describe the lateral position as a function of the UAV longitudinal position for lane change, the polynomial takes a closed form with a continuous curvature:

$y(x)=2r\left\{ 10\left( \frac{x}{d} \right)^{3}-15\left( \frac{x}{d} \right)^{4}+6\left( \frac{x}{d} \right)^{5} \right\}$ (45)

$d_{l}=v\sqrt{\frac{2r}{ẋ_{max}}\left\{ 60\left( \frac{x_{m}}{d_{l}} \right)-180\left( \frac{x_{m}}{d_{l}} \right)^{2}+120\left( \frac{x_{m}}{d_{l}} \right)^{3} \right\}}$ (46)

Where *y*, *x*, *r* and $d_{l}$ are the lateral position, longitudinal position, lane radius, and the target lane change longitudinal distance, respectively. The virtual reference lane is modeled by substituting the required *d* and *r* into equation (45). To optimize the trajectory that does not exceed the UAV’s lateral acceleration limit, the point of maximum curvature ($x_{m}$) based on the trajectory tangents $t(q)$ is computed. Equation (46) determines the appropriate $\delta_{V}, \delta_{H}$based on the overall lane change distance *d* and the maximum UAV lateral acceleration ($ẋ_{max}$) by differentiating $y(x)$ and substituting it.
